# Identification of High-Impact *cis*-Regulatory Mutations Using Transcription Factor Specific Random Forest Models

**DOI:** 10.1371/journal.pcbi.1004590

**Published:** 2015-11-12

**Authors:** Dmitry Svetlichnyy, Hana Imrichova, Mark Fiers, Zeynep Kalender Atak, Stein Aerts

**Affiliations:** 1 Laboratory of Computational Biology, KU Leuven Center for Human Genetics, Leuven, Belgium; 2 VIB Center for the Biology of Disease, Leuven, Belgium; Weizmann Institute of Science, ISRAEL

## Abstract

Cancer genomes contain vast amounts of somatic mutations, many of which are passenger mutations not involved in oncogenesis. Whereas driver mutations in protein-coding genes can be distinguished from passenger mutations based on their recurrence, non-coding mutations are usually not recurrent at the same position. Therefore, it is still unclear how to identify *cis*-regulatory driver mutations, particularly when chromatin data from the same patient is not available, thus relying only on sequence and expression information. Here we use machine-learning methods to predict functional regulatory regions using sequence information alone, and compare the predicted activity of the mutated region with the reference sequence. This way we define the Predicted Regulatory Impact of a Mutation in an Enhancer (PRIME). We find that the recently identified driver mutation in the *TAL1* enhancer has a high PRIME score, representing a “gain-of-target” for MYB, whereas the highly recurrent *TERT* promoter mutation has a surprisingly low PRIME score. We trained Random Forest models for 45 cancer-related transcription factors, and used these to score variations in the HeLa genome and somatic mutations across more than five hundred cancer genomes. Each model predicts only a small fraction of non-coding mutations with a potential impact on the function of the encompassing regulatory region. Nevertheless, as these few candidate driver mutations are often linked to gains in chromatin activity and gene expression, they may contribute to the oncogenic program by altering the expression levels of specific oncogenes and tumor suppressor genes.

## Author Summary

Precise regulation of gene expression is controlled by *cis*-regulatory modules (CRM) containing binding sites for transcription factors (TF). The genome-wide location of all TF binding sites can often be obtained by ChIP-seq (chromatin immunoprecipitation followed by deep sequencing), yet in most cases only a minority of the binding peaks actually represent functional CRMs that control the transcription initiation of a *bona fide* TF target gene. Here, we investigated for 45 cancer-related TFs how machine-learning approaches can be used to predict functional TF target CRMs. After careful evaluation of their performance, we used these TF-target classifiers to predict which *cis*-regulatory mutations may have a significant impact on gene regulation by evaluating whether the mutation causes a significant gain or loss in the probability that the CRM is a functional TF target. We found that Random Forest classifiers can achieve more than 100-fold higher specificity for mutation prediction compared to the simple approaches based on scanning with position weight matrices. By scanning somatic mutations in breast cancer genomes and in the HeLa genome, we finally show that our TF-target classifiers can identify high impact non-coding mutations that are associated with concordant TF binding, gene expression changes and chromatin activity. In conclusion, TF-specific Random Forest classifiers can be used to prioritize *cis*-regulatory mutations in cancer genomes with high accuracy.

## Introduction

Gene regulation determines the identity and behaviour of all cells, and perturbations of gene regulatory programs can cause cells to change their identity or become transformed into cancer cells. Such perturbations of gene regulatory networks can be caused by driver mutations in signalling molecules, transcription factors (TF), and chromatin modifiers [1]. In addition, driver mutations can also occur within the non-coding genomic regions that control transcription, the *cis*-regulatory modules (CRM). CRMs harbour recognition sites for one to many transcription factors and regulate the transcription initiation rate at one or more nearby target genes. Recently two cancer-related CRM mutations have been discovered, namely: a highly recurrent mutation in the *TERT* promoter that is found in many cancer types [2–5]; and a more distally located enhancer mutation upstream of the *TAL1* gene in T-cell acute lymphoblastic leukemia (T-ALL) [6]. These two examples of driver mutations generate *de novo* binding sites for oncogenic transcription factors. Particularly, the *TERT* promoter mutations create new ETS-like binding sites (GGAA), while the *TAL1* mutation creates a MYB binding site. Interestingly, the latter is associated with a very significant gain of the activating histone modification H3K27Ac, indicating that the neomorphic enhancer actively regulates *TAL1* expression.

To analyze *cis*-regulatory variation on a genome-wide scale and to prioritize candidate driver mutations, several types of information can be exploited and integrated [7–11]. A first class of methods is based on *filtering* all candidate variants, such as single nucleotide variants (SNV) and small indels, to retain only those that affect “interesting” nucleotides. For example, a method called FunSeq retains mutations that affect “sensitive” genomic positions (FunSeq also combine other types of data [8]). Sensitive positions are determined by FunSeq as positions that are significantly infrequently substituted in the normal human population. Other methods, like OncoCis [9] and RegulomeDB [11], retain mutations that are located in candidate regulatory regions, as determined by publicly available regulatory data (e.g., from ENCODE [12]). The disadvantage of this approach is that regulatory activity observed in a cancer sample may not correspond to any of the available annotation, particularly when the mutation creates a gain-of-function CRM, or in other words, publicly available regulatory annotation is not always indicative for the function of the CRM in the cancer sample under study. A solution to this problem could be to profile chromatin states in the actual cancer sample itself, but the currently available biochemical methods (mainly open chromatin profiling and ChIP-seq) still require relatively large amounts of input material, which is often not available for tumor biopsies. A second class of approaches is based on QTL analyses, whereby DNA variants are correlated with DNA methylation, chromatin accessibility, or gene expression. These methods have been mostly applied to identify variation in the normal population [13–16] but when larger cohorts of more than 200 cancer samples become available (full genome, methylome, and transcriptome for each sample), they can, in principle, also be applied to identify cancer driver mutations. A related approach is to select mutations that cause allelic shifts in ChIP-seq reads, which was shown to identify functional SNPs that change enhancer activity in HepG2 cells [17]. A third class of approaches, which can be used in combination with the first two, investigates the mutated sequence itself, using information about TF recognition motifs and selects mutations that affect transcription factor binding sites. This can be achieved by scoring the reference and mutated sequences with a position weight matrix (PWM) of a particular TF, assessing the impact of the mutation by the difference of the scores for the reference and mutated sequence. For example, FunSeq calculates “motif maker” and “motif breaker” scores for PWMs and returns a list of all affected PWMs, for each mutation. A limitation of these methods is that PWM-scanning methods are notorious for generating high amounts of false positive predictions, which can affect the accuracy of PWM-based mutation scoring, yielding excessive amounts of false-positive mutations. The prediction of *cis*-regulatory mutations using PWMs would therefore benefit from more advanced models of TF target prediction, so that the impact of a mutation can be assessed more accurately, in the context of an entire CRM. By incorporating CRM context into a predictive model, we may achieve a higher accuracy for predicting functional *cis*-regulatory mutations. When using CRM prediction and classification methods to assess mutations, we can build on a large body of previous methods, using various kinds of features such as TF motifs, other (higher-order or structural) sequence features, sequence conservation, or chromatin related data. CRM prediction methods that are based on motif scanning usually score (sliding) sequence windows for the presence of clusters of TF binding sites, either for the same TF (i.e., homotypic clusters) or for different co-regulatory TFs (i.e., heterotypic clusters) [18–21]. CRM classification methods applying machine learning, using a training set of positive CRMs, are more flexible in terms of the types of features, and once a model is trained, it can be used to predict similar CRMs in the genome. For example, Narlikar et al., employed a Lasso model with a collection of 701 position weight matrices (PWMs), *de novo* discovered motifs and Markov models and were able to predict heart enhancers [22]. kmer-SVM [23] or IMM [24] use a PWM-blind approach whereby the features are entirely learned from the sequence of training CRMs, as over-represented k-mers or Markov chains. Classifiers can also be trained using chromatin data, such as Chromia [25] which uses chromatin data such as histone modification profiles as features in its model, trained on TF binding sites defined by ChIP-seq. It was shown for Chromia that such models, when combined with a PWM, can yield accurate genome-wide predictions of TF targets. More recent methods for enhancer classification use multiple layers of epigenomic data, such as chromHMM [26].

We reasoned that such complex CRM models, when trained on sets of CRMs targeted by specific oncogenic or tumor suppressor TFs, could provide an interesting approach to score putative *cis*-regulatory mutations, and to assess whether the mutation may cause a gain or loss of a functional TF target. To this end, we developed 45 Random Forest classifiers for more than forty different TFs, each trained on subsets of *functional* CRMs (i.e., regions bound by the TF that actively regulate target gene expression). We validate these models by cross-validation and genome-wide scoring, and apply them to identify PRIME mutations (mutations with high PRIME score: Predicted Regulatory Impact of a Mutation in an Enhancer), both using simulated substitutions and real somatic mutations in a large breast cancer cohort from TCGA and in the HeLa genome.

## Results

### Training TF-specific enhancer classifiers to predict functional TF binding regions

**Fig 1:**
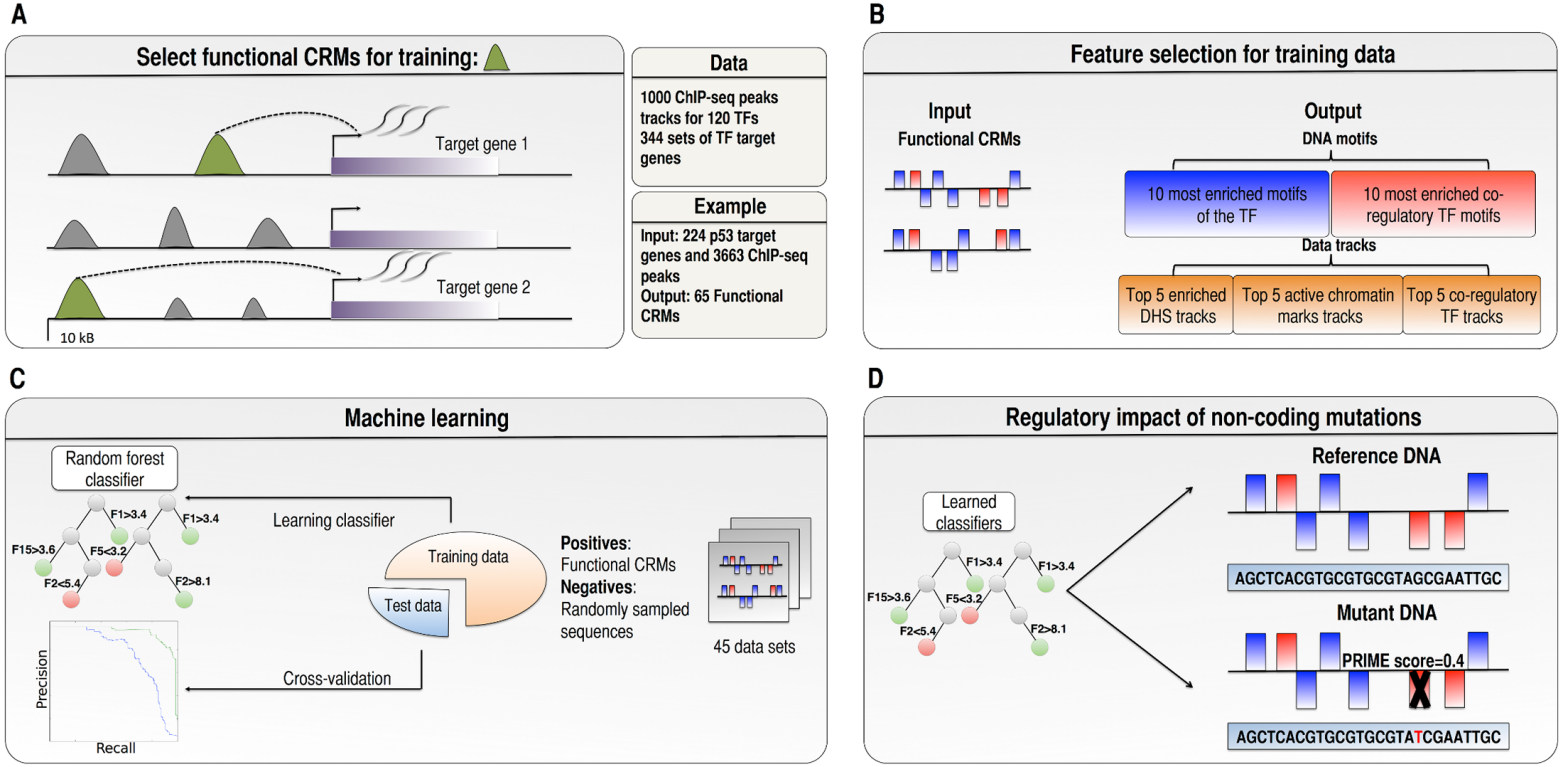
Overview of the methodology. A) To identify functional CRMs we searched for significant correlations between TF ChIP-seq tracks and TF target genes using i-cisTarget [28]; and selected peaks (marked in green) that are located in 20 kb regulatory space around up- or down-regulated TF target genes. B) Feature selection was performed on the set of functional CRMs to select TF and co-regulatory PWMs and data tracks. C) The performance of each of the 45 TF models was evaluated by 5-fold cross-validation, using area under the precision-recall and receiver-operating characteristic curves. D) The 45 learned classifiers where used to identify *cis*-regulatory somatic mutations that have an impact on the CRM score, defining a PRIME score (Predicted Regulatory Impact of a Mutation in an Enhancer).

Chromatin immuno-precipitation coupled with sequencing (ChIP–seq) allows identifying genome-wide locations of TF binding, however usually only a fraction of observed ChIP-seq peaks (0.9%-54.6%) are functional, in the sense of being actively involved in regulating target gene expression [27]. Here, we wanted to develop TF-specific enhancer models by training them only on *functional* target CRMs (Fig 1). To identify such training sets of functional ChIP-seq peaks we searched for peaks that are located near up- or down-regulated genes in response to a perturbation of the TF, or that are located near tightly co-expressed target genes with the TF (see Methods). To obtain statistically significant correlations between ChIP-seq data and co-expressed gene sets, we applied a procedure called “track discovery”, whereby ChIP-seq peak sets from ENCODE and other resources are tested for their enrichment on a gene set [28]. Particularly, we compared 344 sets of TF target genes against 1000 ChIP-seq tracks. This led to the identification of 45 sets of positive training CRMs for 41 distinct transcription factors, most of which are related to cancer. The average size of the training sets ranges between 6 (POU5F1) and 3901 (YY1) positive samples (S1 Table).

**Fig 2:**
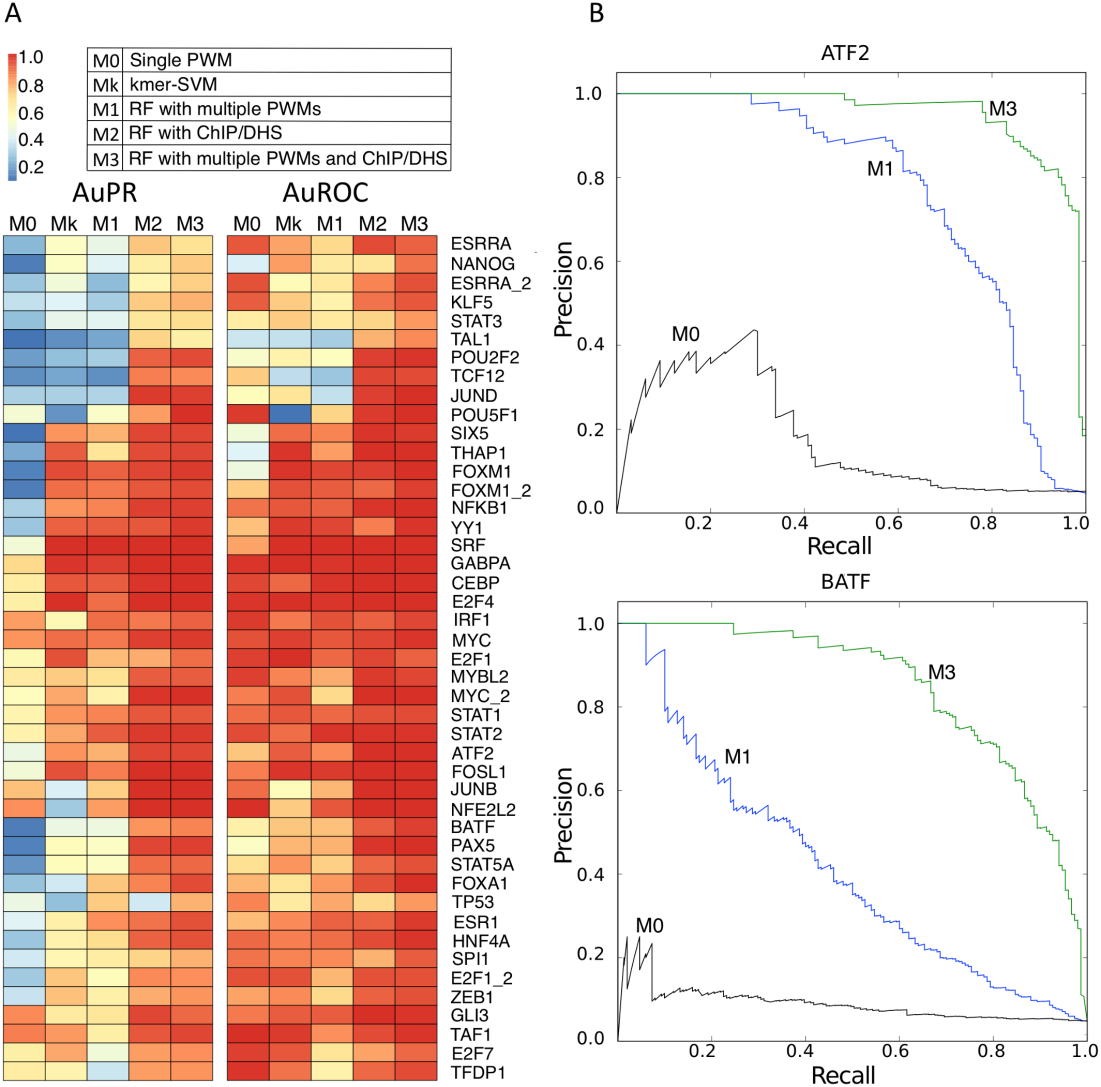
Cross-validation performance for 45 TF models. A) Area under precision-recall (AuPR) and receiver operating characteristic (AuROC) curves for different models. Mk, M1, M2, and M3 are estimated by 5-fold cross-validation. M0 model does not use a training set and the AuROC and AuPR where obtained by varying the threshold of the PWM. B) Examples of precision-recall curves for ATF2 and BATF. Random Forest classifiers outperform PWM-based models. M3 models (using experimental data tracks) outperform M1 models (using sequence only).

For each set of positive CRMs we trained Random Forest (RF) classifiers each consisting of 151 decision trees that optimally distinguish the positive CRMs from sets of negative sequences. As negative sequences we used randomly sampled regions from the human genome with the same size and GC content as the positives, in a 1:20 ratio. We trained different types of RF models depending on the type of features used in the decision trees (Fig 1B). The first model, M1, uses ten motifs of the TF and ten motifs of co-regulatory TFs. These twenty motifs are selected by motif discovery on the training CRMs, out of a collection of nearly 10.000 candidate position weight matrices. The second model, M2, uses as features the fifteen most representative regulatory tracks: five open chromatin tracks, five active histone modification tracks, and five ChIP-seq tracks of potential co-regulatory TFs. Model M3 combines all features of M1 and M2, in total twenty motifs and fifteen tracks. Similarly to motif features, these tracks were selected by track discovery (see Methods). To avoid over-fitting, ChIP-seq tracks of the query TF itself were excluded as candidate features. The performance of each of the 45 TF models (for the three different RF types) was evaluated using the area under the precision-recall (AuPR) and area under the receiver operating characteristic (AuROC) curves, as achieved by the model in a five-fold cross-validation (Fig 2, S1 Fig). We compared the performances to a baseline model (M0) that predicts TF targets by simple PWM-scanning using the PWM of the query TF; and to a previously published alternative classifier based on Support Vector Machines trained on k-mers (Mk) [29]. Collectively, M1 (the RF classifiers utilising only motif information) achieved on average across the 45 datasets an AuPR of 0.62; similar to Mk (kmer-SVM: AuPR = 0.61), and both are much higher than M0 (PWM-only: 0.37). Note that we prefer the AuPR since the AuROC is less reliable for imbalanced training sets with high numbers of negative sequences [30]. The best performing M1 models are for SRF, GABPA, CEBPB, STAT2, and YY1. In total, thirty of the RF classifiers achieved an AuPR greater than 0.5 (Fig 2A, S2 Table). Additional quality control and robustness analysis revealed that most models show stabilization of cross-validation performance (S2 Fig); that Random Forests outperform other machine-learning approaches on the same data such as Support Vector Machines or Logistic Regression (S3 and S4 Figs); and that the performance of the models does not depend on the size of the training set (S5 Fig) nor on the information content of the main PWM (S6 Fig) of the query TF.

**Fig 3:**
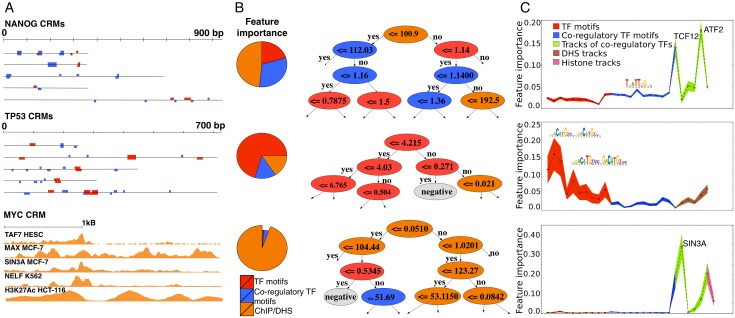
Feature importance. A) Three examples of TFs, each with several (for NANOG and TP53) or one (for MYC) target CRMs, illustrating the feature importance in the Random Forest classifier, in the M3 model. For NANOG co-regulatory PWMs contribute more to the classification performance than the PWM of NANOG itself. For TP53, the contribution of the co-regulatory PWMs is not strong and the classification decision is largely based on the presence of strong binding sites of TP53 itself. For the MYC model the most important features are regulatory tracks. B) Examples of a decision tree in the ensemble. C) Averaged feature importance across trees, showing the contribution of various features to the classification decision. For example TCF12 and ATF2 tracks are dominant for NANOG model; for TP53 the most relevant features are motifs of the query TF (red) and particular important ones are represented with logos. The colored region around dashed line demonstrates standard deviation of the feature impartance across trees.

Next, we investigated the performance of M2 models that use data tracks instead of motifs. These models have drastically higher AuPRs than motif-based models (average AuPR = 0.87), with all 45 models having an AuPR above 0.5. For M2, the best performing models are for SRF, E2F4, JUNB, NFE2L2, and TP53. Interestingly, several TFs with ill-performing M1 models have a much improved AuPR score; for example TAL1 has an M1 model with AuPR = 0.13, whereas M2 with tracks achieves an AuPR of 0.69 (Fig 2A). Finally, for combined models the performance increases even further, although not much beyond M2 (average AuPR = 0.9). Interestingly, TFs can be grouped into different classes, where each class has different types of features contributing to the classifier, as determined by Gini impurity (see Methods) (S7 Fig). For 20/45 models, the TF PWMs contribute more than 20% of all features (e.g., TP53 in Fig 3). For another class of 7/45 models the co-regulatory factor PWMs contribute more than 20% of all features and dominate over the TF PWMs (NANOG in Fig 3); and for 39/45 models the sum importance of the three data tracks groups was dominant providing more than 50% of the feature importance (e.g., MYC in Fig 3).

Fig 3 also shows an example decision tree from the ensemble for the TP53, NANOG, and MYC examples. By investigating the feature importance we can obtain more insight into the CRM code; for example that TCF12 and ATF2 tracks are important to predict NANOG targets; or that SIN3A ChIP-seq peaks in MCF-7 are important to predict MYC binding. Note that this does not mean that SIN3A and MYC necessarily co-bind in the same cell.

In conclusion, we trained multiple well performing models for the classification of TF-specific regulatory target regions. The results suggest that not all information in a CRM can be captured by TF and co-regulatory TF motifs. The track-based M3 models yield an upper-limit to the classification performance based on sequence/motif information alone. Ultimately however, to identify *cis*-regulatory mutations (see further below), we will rely on sequence/motif-based models because those are generally applicable, as they do not depend on the availability of multiple regulatory tracks in the cancer and normal sample.

### Genome-wide prediction of functional TF targets

**Fig 4:**
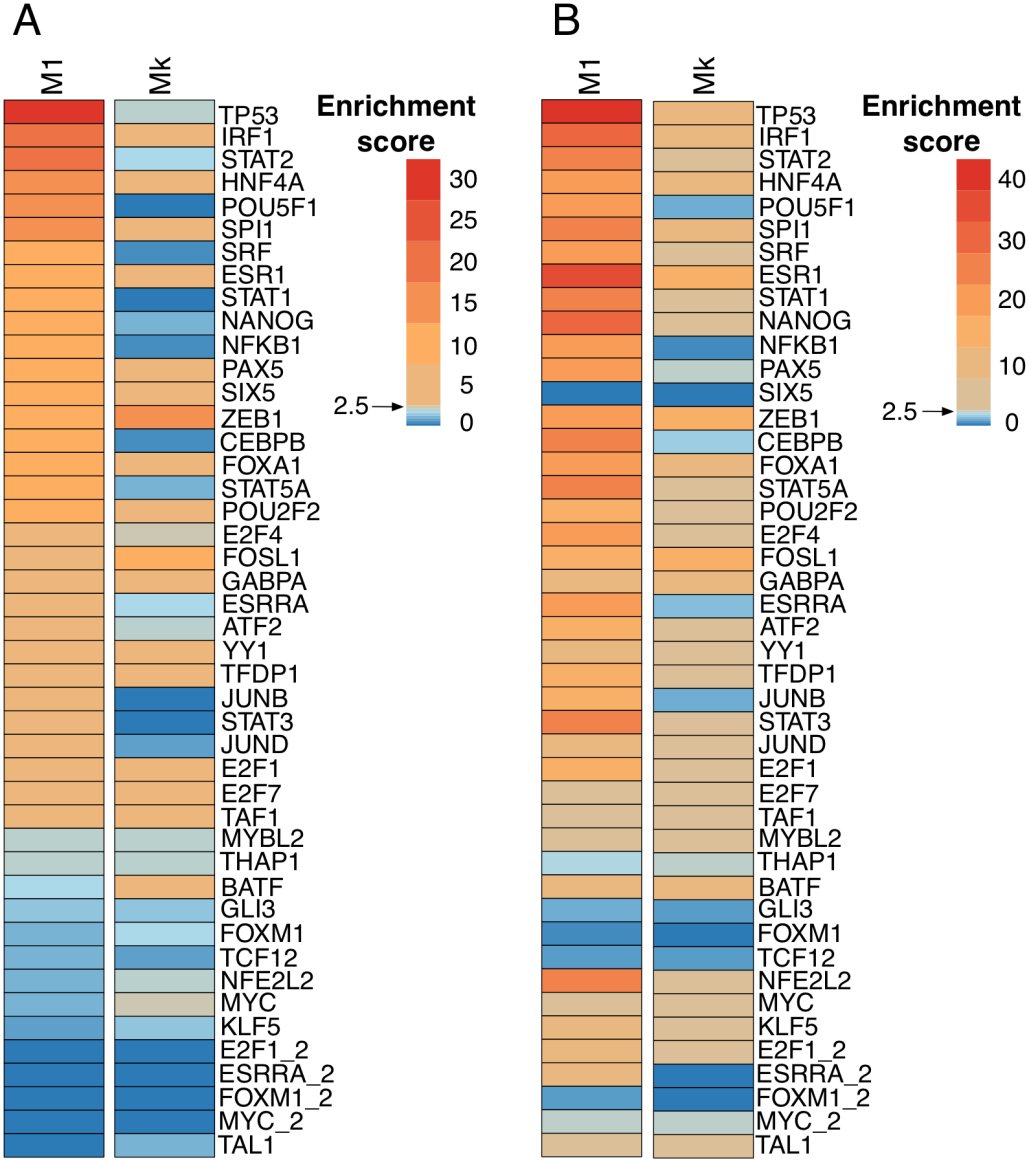
Validation of classifiers by genome-wide CRM prediction. After genome-wide CRM scoring, removing the training CRMs, we evaluated the enrichment of ChIP-seq peaks of the corresponding TF, and the enrichment of motifs of the corresponding TF, within the top 1000 newly predicted CRMs. Enrichment is calculated by i-cisTarget [28], and represented as a Normalized Enrichment Score (NES). A) Significant enrichment of ChIP-seq peaks (orange color corresponds to NES>2.5) for 31/45 M1 models, compared to 17/45 of the Mk models. B) The motif of the respective TF is also enriched in the top 1000 newly predicted functional CRMs, for those in orange (NES>2.5).

To further validate our trained CRM classifers we applied them genome-wide to predict new functional TF target CRMs (including M1, M3, and Mk models). To this end, we split the genome into overlapping sliding windows with sizes corresponding to the average lengths of the sequences in the training set (ranging from 400 bp for NANOG to 2350 bp for GLI3, with an average of 900 bp). The number of newly predicted functional binding sites for M1 models ranges from several hundreds to several tens of thousands. To assess the accuracy of new predictions, we calculated the enrichment of the TF ChIP-seq peaks among newly predicted CRMs, excluding training CRMs. We found a significant recovery for 31 of the 45 models using a RF classifier with motifs only (M1). The five best performing models regarding genome-wide predictions, as measured by the Normalized Enrichment Score (NES) given by i-cisTarget [28], are TP53 (NES = 31.1), IRF1 (NES = 21.5), STAT2 (NES = 17.45), POU5F1 (NES = 16.25), and SPI1 (NES = 14.15). Interestingly, although the cross-validation performances of the motif-only RF and k-mer SVM models were highly similar, the genome-wide prediction accuracies are overall much higher for the RF models (Fig 4A). Particularly, 31 of the 45 M1 models show significant recovery of the correct ChIP-seq peaks, compared to only 17 of the 45 Mk models (Fig 4A). We also performed genome-wide predictions for M3 models, which incorporate regulatory data tracks as features in the model. Although the cross-validation performance of M3 models is much better than M1, the M3 models did not result in more TFs with high-confidence genome-wide scoring, since again 31 models show significant recovery of correct ChIP-seq peaks. This indicates that M1 models with motif-information alone are already very performant in genome-wide predictions, and this is confirmed by inspecting the correlation between the validation scores (i.e., TF track enrichment scores), being very high between M1 and M3 (0.876), while they are both better than Mk (S8 Fig). We also analyzed whether predicted CRMs show enrichment for active chromatin marks, such as H3K27Ac. Indeed, for 26 to 40 models this is the case for M1 and M3 models respectively. More generally, for the majority of TFs (39/45 models) the newly predicted CRMs are enriched for regulatory active chromatin states, as determined by chromHMM segmentations [26] from ENCODE (S9 Fig), with the strongest models overlapping with promoter states being E2F1, TAF1, YY1, E2F7, and KLF5, and the strongest models overlapping with enhancer states being E2F7, TCF12, and FOSL1. In conclusion, we evaluated the quality of the TF-target classifiers in an alternative way, independent of cross-validation performances and found that most RF classifiers are enriched for ChIP-seq peaks of the query TF and active chromatin marks.

### Using enhancer models to predict high-impact *cis*-regulatory mutations

**Fig 5:**
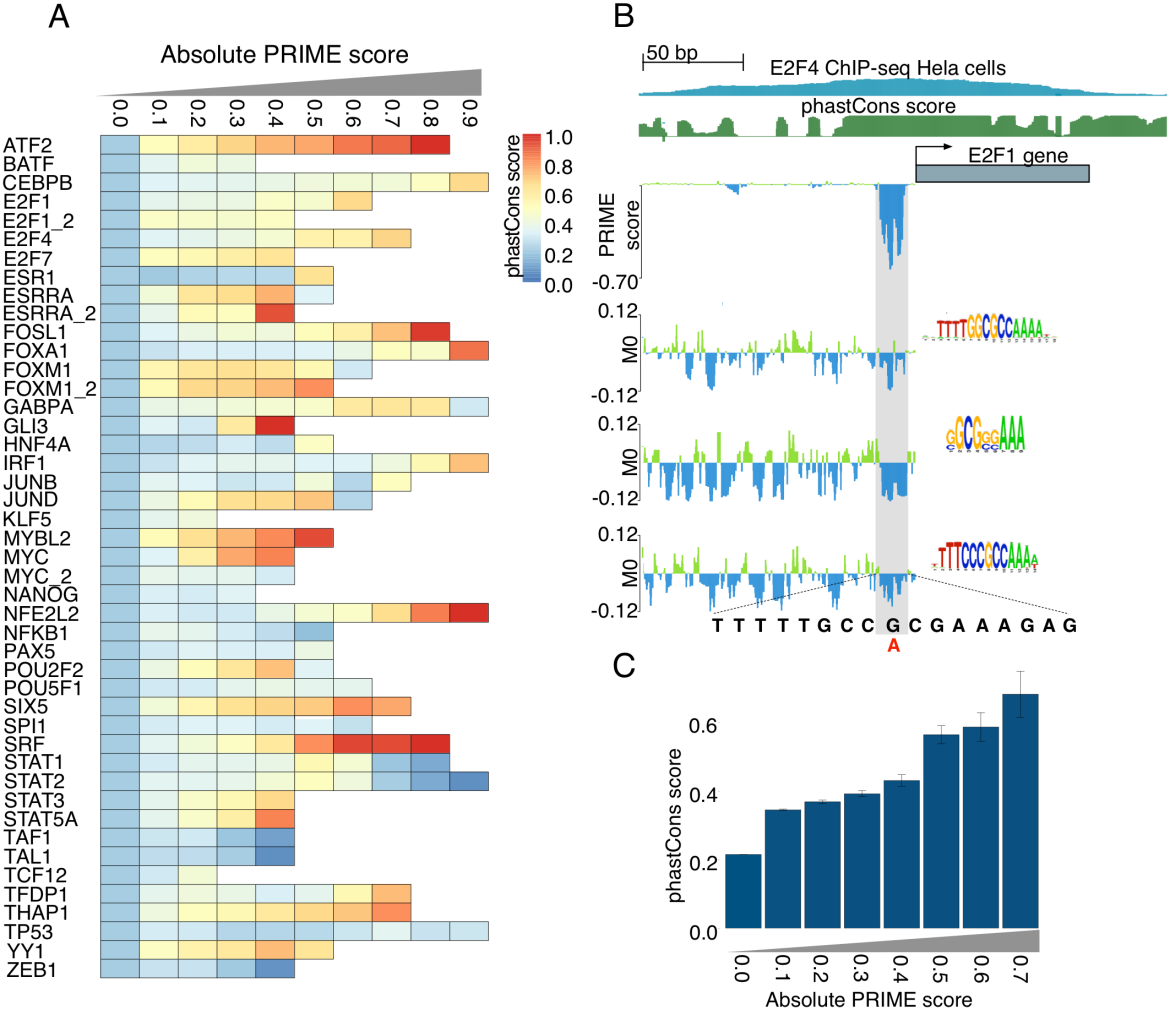
Regulatory impact score on simulated substitions. A) Nucleotide substitutions with higher PRIME scores are under constraint. B) An example of the *E2F1* promoter for which each possible substitution is evaluated by M0 and M1 models. The M1 model (Random Forest) identifies a 15 bp region that is highly vulnerable to mutations, while three different M0 models (using only the PWM), identify excessive numbers of false-positive substitutions, demonstrating the higher specificity of the Random Forest classifiers, compared to single PWMs. C) Barplot showing an example from A), thus averaged phastCons scores depeneding on the PRIME score threshold, for the E2F4 model. Error bars represent standard error of the mean.

Whereas current methods for the prediction of changes in TF binding sites assess local changes in the actual TF binding site, for example using a change in the PWM score [8–10], here we wanted to assess whether TF-specific enhancer models allow identifying *cis*-regulatory mutations that have an impact on the global CRM score. Firstly, we simulated mutations by creating substitutions in gene promoters. Particularly, we selected the 900 bp promoter of 752 curated cancer driver genes [31–35] and changed at each position the sequence into each of the three alternative nucleotides. To measure the impact of each possible single nucleotide variation (SNV) we introduce a score, called PRIME, that is calculated as the difference between the RF classifier scores for mutant and reference sequences. PRIME values range between -1.0 to 1.0 and allow capturing both gains and losses of CRM function. To evaluate the quality of PRIME, we hypothesized that nucleotides with higher PRIME scores should be more conserved. Indeed, nucleotides tend to be under higher constraint with increasing absolute PRIME score (Fig 5A). There is one caveat to this analysis however: low PRIME scores can represent a mixture of sites that are not bound by either allele, and bound sites where the variant does not change binding. To distinguish between these, we simulated substitutions inside ChIP-peaks (true sites) versus substitutions outside ChIP-peaks (not bound by either allele) in terms of conservation (S10 Fig). The results demonstrate that although nucleotides belonging to real binding sites tend to be more conserved with increasing PRIME score, also high-scoring mutations outside ChIP peaks are enriched for high phastCons scores. We performed a similar validation experiment using open chromatin data and found that substitutions with high PRIME score tend to be more located in accessible regions than low PRIME substitutions, suggesting their potential involvement in CRM function (S11 Fig). As an example, we show in Fig 5B the promoter of the *E2F1* gene, where the E2F4 model identifies a hotspot of high PRIME substitutions. Convincingly, these positions overlap with the summit of an E2F4 ChIP-seq peak and cover the entire E2F4 consensus site. We expected an increased specificity (rather than sensitivity) of mutation detection with Random Forest models (M1) compared to the simple PWM model (M0), because PWMs are known to suffer from high false positive rates [36]. To test whether this is indeed the case for the *E2F1* promoter, we scored all possible substitutions in this promoter with several E2F4 PWMs, and indeed found many non-functional positions that show a change in PWM score (Fig 5B). This suggests that random forest classifiers are better suited to detect *cis*-regulatory variation than PWMs.

We then scored a large collection of real non-coding somatic mutations collected from three cancer whole genome sequencing studies: 50 AML samples (N = 19797) [35], 21 breast cancer samples (N = 183703) [37], and 25 melanoma samples (N = 1875157) [38]. Similarly to the simulated substitutions, we found that predicted high-impact mutations are more conserved than mutations with low predicted regulatory impact (S12 Fig). Also, mutations with high absolute PRIME score (greater than 0.4) are enriched for chromatin states corresponding to functional regulatory elements such as active promoters, weak promoters, and strong enhancers (S13 Fig). When compared to measuring the impact of a mutation by the change in PWM score, also on this set we find that the Random Forest models show greater specificity than PWMs (S14 Fig).

In conclusion, the TF specific classifiers can identify regulatory variation affecting the activity of functional CRMs, making this a feasible strategy for the prediction of cancer driver mutations.

### Previously known driver mutations in the *TAL1* enhancer are predicted as high-impact *cis*-regulatory mutations

To test whether the Random Forest CRM models may be suitable to identify cancer driver mutations we examined in detail a recently published *cis*-regulatory mutation in the *TAL1* promoter in T-cell Acute Lymphoblastic Leukemia (T-ALL) [6]. Particularly, a recurrent (5.5% of patient T-ALL samples) mutation is caused by a short insertion that creates one or two *de novo* binding sites (depending on the length of the insertion) for the MYB transcription factor, a well-known regulator involved in T-ALL. Our 45 models do not contain a MYB-specific model (only a MYBL2 model), and none of the 45 models predicted a high PRIME score for this site. However, when we trained a MYB-specific M1 model, using MYB target CRMs as training set (obtained by anti-MYB ChIP-seq in the Jurkat T-ALL cell line [6]), the *TAL1* mutation yields a very high PRIME score (from 0.054 in the reference to 0.3774 in the mutated CRM). Thus, only the MYB model identifies this gain-of-function mutation. In contrast, when we used the PWM for MYB, which yields an increase in PWM score of 0.1 for the actual driver mutation compared to reference (from 0.844 to 0.949), we also find two other PWMs of the 45 tested M0 models (GABPA and CEBPB) that yield a similar PWM score increase (more than 0.1) and that have a high PWM score (>0.9) for the mutant sequence. In other words, although the MYB PWM can identify the mutation, it is also falsely predicted by other PWMs, but not by other Random Forest models.

**Fig 6:**
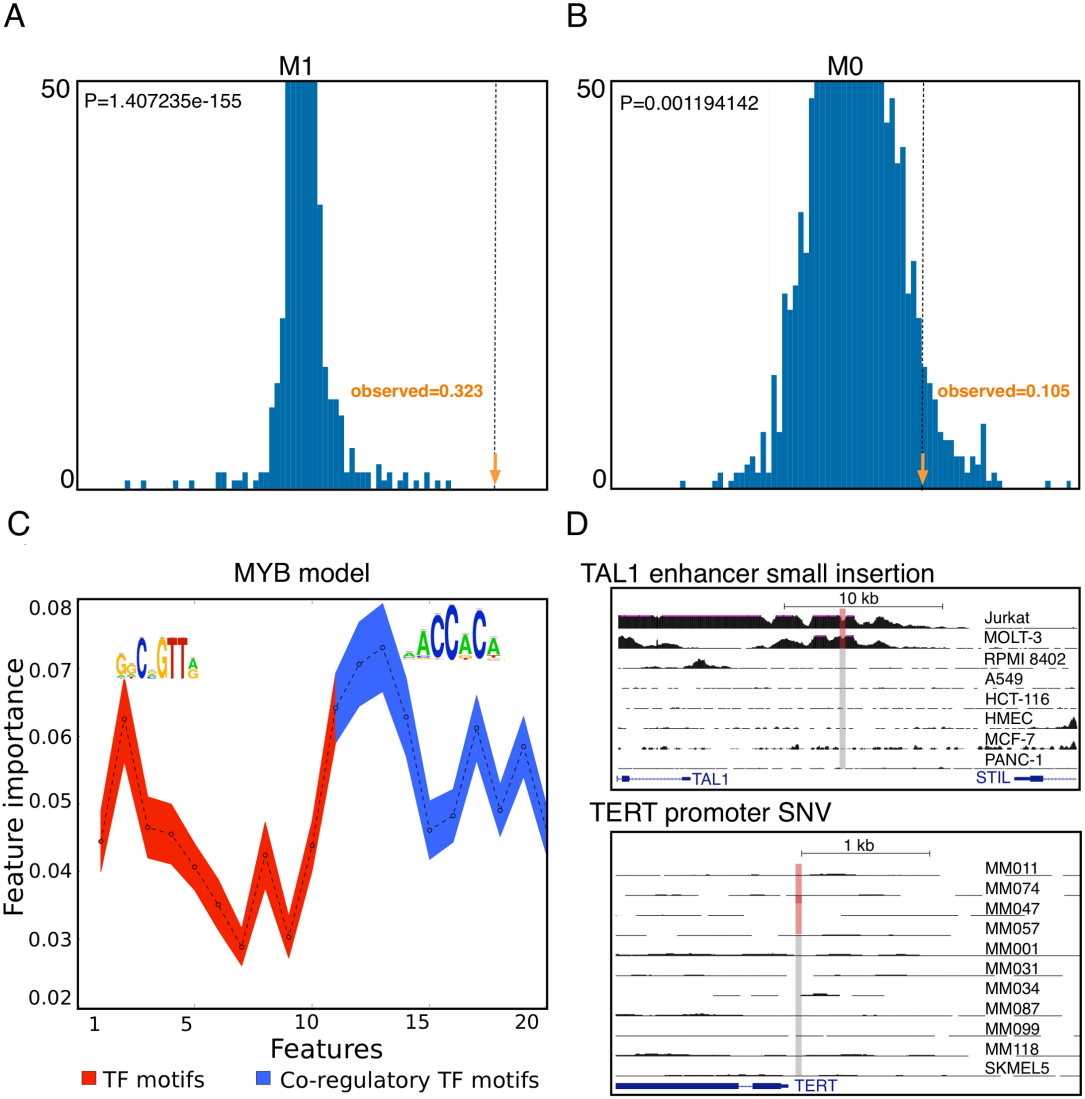
Comparison of PWMs and Random Forest classifiers on the known *TAL1* insertion. We scored the known *TAL1* enhancer insertion that occurs in the Jurkat cell line [6] with Random Forest (M1) and PWM (M0) MYB-specific models. As control, we scored all SNVs and insertions in promoters across 498 breast cancer genomes with the same MYB models, to calculate a background distribution of impact scores. A) The distribution of background PRIME scores (i.e., delta Random Forest scores) and the observed PRIME score for M1, indicated as the orange arrow. B) The distribution of background PWM-delta scores (M0 model) and the observed score. C) Feature importance within the MYB model indicates that both and MYB motifs and co-regulatory TF motifs contribute significantly to the classification decision and the most important co-regulatory motif is RUNX, a known co-regulatory factor of MYB. D) The known driver insertion in the *TAL1* enhancer generates a gain of H2K27Ac peak, whereas the known SNV in the *TERT* promoter does not. The red highlighted region indicates which samples harbor the respective *cis*-regulatory mutation.

For a MYB model to prioritize this mutation in the genome, out of all possible somatic mutations, the model also needs to be specific. To test this, we scored a large set of control somatic mutations (both SNVs and insertions) with the same MYB model (Fig 6A and 6B). These control mutations were selected from breast cancer somatic mutations from TCGA. Since MYB is not known to be involved in breast cancer, we could argue that each mutation with a high PRIME score for the MYB model would be a false positive prediction. This analysis shows a remarkable specificity, with only 2/19796 SNVs and 0/7323 insertions predicted as high-impact mutation for MYB (PRIME>0.3). For comparison, using the MYB PWM identifies 179 SNVs and 354 insertions with a delta of 0.1 or more in the control set. For the *TAL1* promoter mutation we can conclude that the predicted high impact corroborates the gain of CRM activity observed in the Jurkat cell line that harbors this mutation, as measured by H3K27ac (Fig 6D).

The empirical distribution of background PRIME scores for the MYB model allows estimating the significance of this PRIME score using a z-score (see Methods), which is 26.5 for the Jurkat insertion. A similar but shorter insertion was found in the MOLT-3 cell line and in several patient samples; these insertions generate only one new MYB binding site and yield z-scores between 1.41 and 21.45 for the MYB model. Note that we used these thresholds based on the MOLT-3 insertion, determined from the empirical distribution of PRIMEs for SNVs or insertions thresholds (9.65 and 14.03, respectively) to determine model-specific PRIME thresholds for other models, further below.

To investigate why the Random Forest model for MYB achieves such high specificity compared to the PWM, we analysed the feature importance within the MYB model and found that both MYB motifs and co-regulatory TF motifs contribute significantly to the classification decision. Interestingly, the most important co-regulatory motif is RUNX, a known co-regulatory factor of MYB (Fig 6C). The combination of MYB motif clusters and co-regulatory motifs allows assessing the impact of a mutation taking the context of a CRM into account. To illustrate this, we tested whether inserting exactly the same sequence at random position does not always produce a similar gain of function. Indeed, when we inserted the same sequence into 100 randomly chosen genomic loci having the same 3 bp flanking nucleotides we found that the PRIME score strongly depends on the surrounding sequence context. For example, the Jurkat insertion generates a PRIME score equal or higher than 0.32 (the observed PRIME in the *TAL1* enhancer) in only 10/100 locations, indicating that most genomic locations are not susceptible to this insertion, in terms of MYB-dependent activity (S15 Fig).

We also performed this analysis for another well-known promoter mutation, in the *TERT* promoter. The *TERT* promoter harbors two recurrent mutations and these are among the highest recurring mutations in cancer (between 33% and 85% in melanoma [2]). The original articles reporting this mutation suggested that this mutation generates an ETS-like binding site (GGAA) and that ETS family members might cause an up-regulation of the *TERT* gene due to this gain of function binding site mutation. More recently, these mutations were linked to *de novo* binding by GABPA, which also binds to a GGAA motif [39]. However, our GABPA model did not result in a significant PRIME score (PRIME = 0.026; Z-score = 0.99). We constructed four alternative models for different ETS-like factors using their respective top ChIP-peaks as training set (see Methods), namely ELF1, ELK1, ELK4, and ETS1. For two of these models, namely for the EFL1 and the ELK1 model, we found significant PRIME scores (z-score = 2.83 and 6.49, respectively), although the PRIME score was much lower than for the *TAL1* mutation (the highest PRIME is 0.097 for ELK1 = >*TERT*, compared to 0.32 for MYB = >*TAL1*). Remarkably, looking at promoter activity data by H3K27Ac, across a cohort of melanoma samples we generated before [40], we could not observe any gain of activity in the samples that harbor the mutation (Fig 6D). We can conclude for the *TERT* promoter that the predicted impact scores are significant but modest and that they corroborate with low observed impact at the promoter activity level.

### Identification of *cis*-regulatory mutations linked to gene expression and chromatin activity

**Fig 7:**
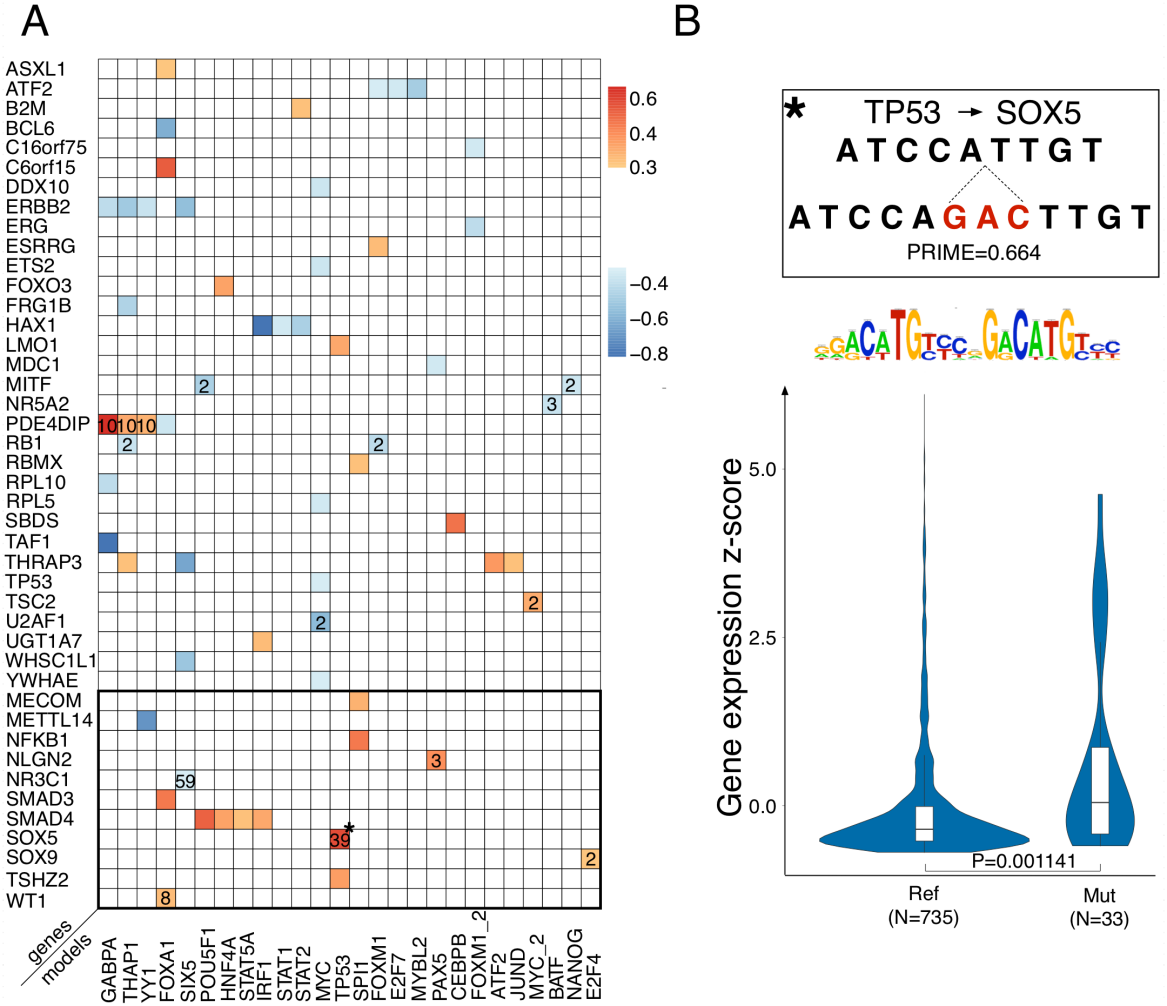
Candidate *cis*-regulatory driver SNVs and insertions across 498 breast cancer genomes. A) All SNVs and insertions with high PRIME score (>0.3) (insertions are within the black box) found by M1 models in the regulatory regions around cancer related genes and 167 TFs expressed in breast cancer (all significant PRIME scores with model-specific thresholds are provided in S5–S6 Tables). Values inside boxes indicate the recurrence, that is the number of samples where this variant was found across the 498 TCGA samples. B) An example of a high scoring recurrent insertion that is predicted to generate a TP53 gain of target in the vicinity of SOX5. Z-scores of the SOX5 gene expression are significantly higher (Wilcoxon rank sum test) in the 33 samples with the insertion, compared to samples without the insertion.

Next we used the TF-specific random forest models to prioritize *cis*-regulatory mutations in 498 re-sequenced breast cancer genomes from TCGA, for which gene expression data is available [41]. We specifically scored all SNVs and insertions located in promoters (see Methods). To evaluate whether mutations with high PRIME scores could have a functional impact on gene expression, we evaluated the expression level of the target gene in the sample with the mutated promoter, compared to all other samples (using z-scores). Indeed, this shows a clear association of changes in gene expression with predicted impact of promoter mutations (S16 Fig). Moreover, the median absolute z-score values of gene expression increases with increasing PRIME score. When we focused on promoters of cancer related genes (the list of 752 curated cancer driver genes), we found only 36 genes having single nucleotide mutations with absolute PRIME score > 0.3 (Fig 7A, S3 Table). Using the model-specific z-scores (with a cutoff of 9.65 for SNVs and 14.03 for insertions), 84 genes are found with significant mutations. When we applied our models to small insertions in promoters, we found only three high impact insertions, namely in the *SOX9* promoter (gain for E2F1), the *METTL14* promoter (YY1 loss), and the *NLGN2* promoter (PAX5 gain). Interestingly, two of these three mutations are recurrently mutated across the TCGA cohort (Fig 7A). Expanding our search to 10 kb, and focusing only on breast-cancer related transcription factors as targets (along the lines of the MYB-*TAL1* gain), we found an additional 91 SNVs and 11 insertions with high impact (S4 Table), including a gain of TP53 CRM upstream of *SOX5*, and a loss of a SIX5 site upstream of *NR3C1*. Interestingly, these two latter insertions are recurrent across the TCGA cohort (39 and 59 samples, respectively). Furthermore, expression of *SOX5* target gene is significantly higher in the samples with the insertion, compared to the samples without the insertion (Fig 7B). Overall, we thus found a limited number of potentially harmful *cis*-regulatory mutations, given that in Fig 7A we pooled together all the results across 498 breast cancer genomes.

**Fig 8:**
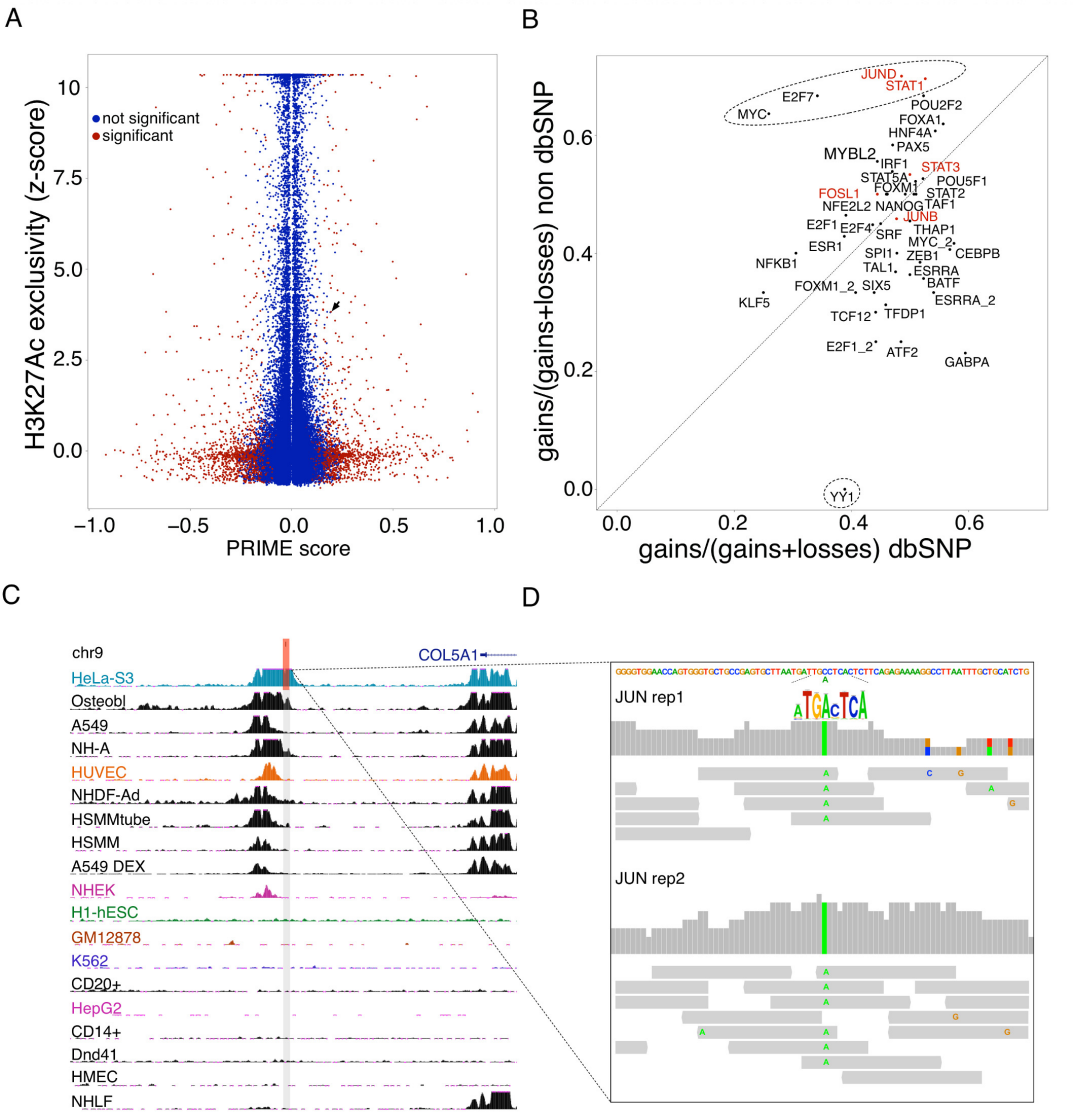
Scoring *cis*-regulatory variants in the HeLa cell-line. A) Scatter plot of PRIME scores (45 M1 models) for heterozygous SNVs in the HeLa cell line versus z-scores of H3K27Ac peak scores (the higher the z-score the more exclusive the H3K27Ac signal to Hela, compared to 108 other samples). The arrow indicates an example SNV that generates a *de novo* JUN binding site (shown in C-D). B) Using high-scoring SNVs falling in acetylation peaks for each TF model we plotted fractions of gains and losses in dbSNP (polymorphisms) versus not in dbSNP (possibly somatic mutations). Oncogenic TFs that are important for HeLa, namely MYC, E2F7, JUND, and STAT1, have more gains than losses, specifically for variations not in dbSNP. *Vice versa*, YY1, a known repressor related to cancer, has almost no gains in non-dbSNP variations, while dbSNP variations have an almost equal amount of gains and losses. C) H3K27Ac signal around SNP that is predicted to generate a gain in JUN binding (PRIME = 0.21; z-score = 16.28) indeed shows a moderate exclusivity of H3K27Ac to HeLa. D) This position shows an allele-specific binding of JUN, only having ChIP-seq reads with the variant allele that causes a gain in JUN binding sites.

Finally, we reasoned that if a mutation really causes a gain of CRM activity, this should be directly visible as a change in chromatin activity, such as increased chromatin accessibility, increased H3K27Ac signal, or decreased DNA methylation. Unfortunately, none of these data are available at the genome-wide level for the TCGA cohort (DNA methylation is currently available for 450K probes, which is too sparse for our low number of high-impact mutations). To test a potential correlation between mutations with high PRIME scores and chromatin, we therefore used the HeLa genome [42,43], for which H3K27Ac data is available from ENCODE (GSM733684) [12]. Scoring all 13923 small insertions located in 10 kb regulatory space around TSS of the HeLa genome, for our 45 models, we found 141 variations with significantly high PRIME scores, based on the model-specific z-scores (S17 Fig). A small subset of these are indeed located in regions with H3K27Ac signal that is specific, or semi-specific for the HeLa cell line (compared to H3K27Ac data for 108 other samples, see Methods), possibly indicating that these mutations have a local effect on the activity of the enhancer (Fig 8A). To test whether any particular TF has more mutated CRMs, we compared the amount of gains and losses for each TF model stratified on whether the variation is a known polymorphism from dbSNP or not, the latter representing possibly somatic mutations (Fig 8B). Interestingly, this shows that oncogenic TFs that are important for HeLa, namely MYC, E2F7, JUND, and STAT1, have more gains than losses, specifically for variations not in dbSNP. Vice versa, YY1, a known repressor related to cancer, has almost no gains in non-dbSNP variations, while dbSNP variations have an almost equal amount of gains and losses. We believe that such skews towards “relevant TFs” strongly indicate a *cis*-regulatory effect for this group of mutations. AP-1 (JUN/FOS dimer) is indeed a relevant factor for HeLa, because it is the most enriched motif and track among all the HeLa-specific H3K27Ac peaks (AP-1 motif rank = 1, NES = 7.36; FOS ChIP-seq NES = 7.20). A clear example of a SNP with a *cis*-regulatory effect is shown in Fig 8C, where a heterozygous SNP that is predicted to generate a gain in JUN binding (PRIME = 0.21; z-score = 16.28), indeed shows a gain of JUN binding in the HeLa ChIP-seq data for JUN (Fig 8D). All the reads in the JUN ChIP-seq peak contain the alternative (non-reference) allele, which generates an AP-1 binding site in a favorable CRM context. Note that compared to the MYB-*TAL1* interaction (see above), which generates a *de novo* super-enhancer that is unique to the Jurkat and MOLT-3 samples, for the HeLa genome we did not identify such strong effects in H3K27ac gains. Indeed, only four insertions are located in a H3K27Ac peak that is unique to HeLa. One of these four has an absolute PRIME score close to 0.3 (-0.295) (i.e., observed frequency is 0.25). Interestingly, this predicted mutation is located near *CDH10*, a gene that is specifically expressed in HeLa, compared to other cell types of the human body map, as determined by Landry et al. [43] (S17 Fig).

In conclusion, TF-specific random forest classifiers can identify *cis*-regulatory variation with potential impact on the function of a promoter or enhancer.

## Discussion

Whole genome sequencing of cancer samples has revealed that cancer genomes harbour thousands to hundreds of thousands of non-coding mutations. Sifting through all these mutations to identify mutations that contribute to the oncogenic process is a key challenge in cancer genomics, as it is yet unclear to what extent regulatory mutations can be actual driver mutations. For coding mutations, driver mutations are usually identified by their significant recurrence across a patient cohort. For example, *TP53* is mutated in 37% of breast cancer samples in the TCGA and Sanger cohorts [41,44]. Thus far, although non-coding mutations are more numerous than coding mutations, very few recurrent *cis*-regulatory mutations have been found, and recent pan-cancer analyses concluded that in fact only one potential *cis*-regulatory mutation, in the *TERT* promoter is highly recurrent [2,3]. *TERT* promoter mutations have been identified in 6 of 14 cancer types where they occur in 3 to 62% of cancer samples, depending on the type of cancer [45]. They are associated with higher expression of *TERT*, both in promoter-luciferase assays [3], and in patient samples [46]. Because these mutations generate GGAA sites, it was hypothesized that this could lead to increased activation by TFs of the ETS-family, which recognize GGAA consensus sites. Recently, it was shown that the TF is in fact GABPA. While the *TERT* mutation has relatively low PRIME scores and no gain of H3K27Ac, the *TAL1* promoter mutation, which generates a *de novo* MYB binding site, causes neomorphic/ectopic enhancer activity as seen by a very strong and broad H3K27Ac signal spanning a large region encompassing the mutation. Interestingly, our enhancer models for MYB predict the *TAL1* mutation as a high-impact mutation.

We have applied CRM prediction methods to the reference genome and to cancer genomes, and calculated the differential CRM score between the reference sequence and the sequence carrying a single nucleotide variant or a small insertion. CRM prediction methods are computational techniques to predict regulatory regions (e.g., promoters, enhancers) based on their sequence content and usually take advantage of transcription factor motifs [47,48]. Whereas CRM prediction methods have often been applied to identify tissue-specific enhancers (e.g., human heart enhancers [22], Drosophila tissue-specific enhancers [24], etc.), their application to identify TF-specific target CRMs is relatively limited [49–51]. Here we specifically train models on training sets of *functional* TF ChIP-seq peaks. We define functional peaks as the signficant subset (or “leading edge” [28]) of peaks that are located near genes that are up- or down-regulated upon perturbation of the TF. Compared to previous methods that often rely on k-mers, Markov chains, or *de novo* discovered motifs in the training set [24,29], we have here assessed the power of using large PWM collections. Since we know (to a large extent) the identity of the TF for each PWM, this strategy allows selecting a set (we choose 10) of specific PWMs for the query TF, and a set of PWMs for potential co-regulatory TFs. We furthermore believe that the power of using PWMs for CRM predictions will further increase, given the recent progress in high-throughput determination of TF binding specificities [52]. Interestingly, we found that for a subset of TFs the co-regulatory transcription factor motifs have a higher feature importance than the motifs of the query TF itself. An important example of this category is the cancer-related TF FOXM1, which requires a Random Forest model with co-regulatory factor motifs to identify FOXM1 targets in the genome. This is also corroborated by the fact that FOXM1 ChIP-seq peaks are not enriched for any FOXM1 motif [53]. Therefore, when potential *cis*-regulatory mutations are scored for their potential motif-breaking or motif-making effects, using the FOXM1 motif would render meaningless results.

As an alternative we have also trained CRM models using regulatory data as features, such as histone modifications and chromatin accessibility. Corroborating previous work by others [25,49,54], such models have a higher performance compared to sequence-based prediction methods. This likely implies that CRM function/output cannot entirely be captured by sequence and motif content of the CRM itself. In this respect, we consider the models using experimental regulatory data to represent an upper limit to the CRM prediction problem. Although for some TFs the sequence-based models reach an accuracy close to their respective data-based model, the performance of most TF models is still far from perfect (15 TFs with AuPR<0.5). To evaluate our models and to compare different approaches we used standard cross-validation. Importantly however, we included a complementary evaluation approach, namely the genome-wide prediction of CRMs. We then tested the performance of each model by assessing the overlap between predicted TF-specific CRMs and TF ChIP-peaks, excluding the ChIP-peaks used in the training set. This allowed to functionally validate our predictions, and to compare our models with alternative modelling approaches (namely, a simple PWM, a k-mer SVM approach, and a gapped k-mer SVM), showing that Random Forest classifiers outperformed these alternative methods (S18 and S19 Figs). In addition, this experiment showed that the predicted CRMs using sequence-based models represent regions with typical characteristics for CRMs, such as cross-species conservation and enhancer/promoter-related chromatin states, including DNAse I hypersensitivity and H3K27Ac enrichment.

Encouraged by the high CRM prediction performance, we then applied the optimized TF-specific CRM models to mutated cancer genomes, to predict *cis*-regulatory mutations with potential impact on CRM function. Using thresholds of the PRIME score based on a z-score, which is calculated on a TF model-specific empirical distribution, by scoring that model on 20000 variants from TCGA; we found relatively few mutations, with only 0.1%– 1.2% high impact mutations with PRIME>0.3 (comparable to the MYB-*TAL1* mutation) per cancer genome, on average. This was true for the large TCGA breast cancer cohort, but also for smaller cohorts of melanoma (25), another breast cancer (21), and AML (50) genomes. Nevertheless, the high-scoring mutations, as well as simulated substitutions we introduced in promoters of cancer genes, overlap significantly with conserved nucleotides and with enhancer/promoter chromatin states, indicating that these predictions are valuable. The low number of high-impact mutations was again confirmed when we scored insertions found in the HeLa genome (scoring 10 kb regions around TSS) [42,43], where we found only one insertion (near *CDH10*) with high impact and associated with a gain in H3K27Ac signal (as the reported insertion in the *TAL1* enhancer). Note that although the thresholds we have applied to PRIME scores are based on model-specific z-scores, the stringent z-score cutoffs are largely inspired by the TAL1 examplars, and could be fine-tuned or relaxed in the future if more experimentally validated cis-regulatory mutations are discovered. Another reason why we identify few mutations may be partly due to the limited number of models we have built (currently 45; with an additional nine models specifically added for the HeLa genome), but we speculate that even with more models, the total number of high impact *cis*-regulatory mutations will be low. This is indeed corroborated with the low number of *cis*-regulatory mutations that are found to be recurrent across cancer samples [7,45]. Importantly, when PWMs are used to score *cis*-regulatory mutations, more than 100-fold excess of false-positives are predicted. This is mostly due to the context of the CRM, for example, when multiple binding sites of the same factor are present in a CRM (i.e., a homotypic cluster [55]), adding or deleting a single binding site may not have any dramatic effect on the CRM. This is measured in the first layer by the CRM score, but not by the individual PWM scores. In a second layer, different features are combined via optimized parameters in an ensemble of decision trees, further increasing the specificity (S20 Fig). Recently, a similar method was published, called deltaSVM [56], which also uses a machine-learning approach to train a model and score reference and variant sequence, to calculate a delta score. Although deltaSVM was mainly applied to GWAS data to score natural variation, it could in principle also be applied to cancer mutations. This method is complementary to our Random Forest models because it is trained on a different type of training set (all open chromatin regions of a sample, rather than TF-specific models) and it uses entirely different features (k-mers for the deltaSVM, compared to PWMs and data tracks for our PRIME scores). Therefore these two approaches are complemenatary and both can predict the impact of mutations in an enhancer, as shown in S21 Fig, on a data set of synthetic enhancer sequences. Nevertheless, since our RF models are often more specific in genome-wide scorings, they may also yield less false-positive predictions on genomic variation (S22 Fig). A future challenge will be to use M3 models to score mutations, incorporating epigenomic data tracks into the model. To this end however, besides a fully re-sequenced cancer genome and germline control, also a cancer and control epigenome would be required. As an alternative, if full genomes can be phased into haplotypes, M3 models could be exploited to score the allele-specific impact of heterozygous variants.

In conclusion, we presented an approach to model *CRM context* allowing to predict and prioritize candidate *cis*-regulatory mutations in cancer genomes that could affect CRM function, and provide a solution to the excess of false-positive predictions obtained by approaches using position weight matrices. Our predictions on cancer genomes furthermore suggest that the majority of non-coding mutations may be passenger mutations, and that only few top-scoring mutations may contribute to the oncogenic program as *cis*-regulatory drivers.

## Materials and Methods

### Selection and identification of TF target genes

TFs target genes were selected either from curated TF perturbation gene signatures (MSigDB) [57] or from a GENIE3 [58] inferred co-expression network focused on melanoma (skin (77): GSE7553 [59], GSE28914 [60], GSE13355 [61]; primary melanoma (90): GSE7553 [62], GSE19293 [63], GSE23376; metastasis (71): GSE7553, GSE10282, GSE22968 [64]. As parameters of GENIE3 we used as input list of transcription factors 2245 items (combined from TRANSFAC^®^ Professional database and MSigDB collection (v4.0)), and a threshold of 0.005 (2041 regulatory modules were identified). In total, we selected 224 curated sets and 120 predicted sets based on the availability of TF ChIP-seq data.

### Identification of a subset of functional ChIP-peaks

For each target gene set we performed “track discovery” using i-cisTarget [28], on all available TF ChIP-seq tracks. If the corresponding TF ChIP-seq track was significantly enriched, the leading edge was selected as optimal target CRMs. As parameters of i-cisTarget we used a search space size of 20 kb around TSS. For four TFs (E2F1, FOXM1, ESRRA, MYC) we found two different studies that provided a target gene set for this TF and for which i-cisTarget found the ChIP-seq of the factor enriched, thus for which we could identify a training set of functional target CRMs. These models are named as E2F1_2, FOXM1_2, ESRRA_2, MYC_2.

### Additional models for TERT and TAL1 mutations

Besides the 45 models trained using the high-throughput procedure above, we trained a MYB model using the top 500 peaks from ChIP-seq data from [6] and several models for ETS-like factors, namely ELK1, ELF1, ELK4, and ETS1, each time using the top 500 ChIP-seq peaks from the ENCODE data [12].

### Feature selection

To select DNA motifs and regulatory tracks enriched in the set of training CRMs we again used i-cisTarget, but now using regions as input. i-cisTarget uses a large collection of motifs (9,713 PWMs) and human regulatory tracks (2,046) derived from different resources [28]. Two groups of motifs where selected: the top ten enriched motifs of the query TF and the top ten motifs of co-regulatory TFs. In addition, for M2 and M3 models, three groups of the most representative regulatory tracks were selected: up to five open chromatin tracks, five histone modification tracks (active marks), and five ChIP-seq tracks of potential co-regulatory TFs selecting only enriched tracks.

### Cross-validation

We performed 5-fold cross-validation. The selection of features using i-cisTarget was performed only once, on the entire training set. This does not affect the cross-validation performance because this filtering step is performed in an unsupervised way (without using the negative samples) [65]. We confirmed this by performing i-cisTarget on every fold, without using the left-out samples, thereby using different features during each fold, but as expected this had no influence on the the AuPR values for cross-validation (S23 Fig). Note that for small training sets (e.g., POU2F1 has only 6 positive CRMs in the training set, the 5-fold cross-validation leaves out only 1 or 2 samples, thus making it more a leave-one-out cross-validation.

### Feature-vector representation of the DNA sequence and Random Forest

Selected enriched PWMs and tracks were used for numerical representation of the DNA regions. For the motif scores we used Cluster-Buster (with default parameters except option -c was set to zero to obtain a score for every sequence) employing a Hidden Markov Model to score CRM sequences for clusters of binding sites [18]. We consider the PWMs as features and for each PWM we calculate on a CRM (which is a sample, so positive or negative) the total Cluster-Buster motif-cluster score for that PWM. This means that for each feature (PWM) we have one score per CRM (so per window). The final M1 models thus contain only 20 features, and each region’s feature vector contains 20 Cluster-Buster scores. For M2 and M3 models we also include data tracks as features. For their scores we assigned the maximum score of broad or narrow peaks (corresponds to signalValue column in the bed file format) overlapping with the scoring region (the overlap was obtained using BEDtools [66]). As negatives we used 20x more sequences, randomly selected from the genome without restriction on genomic locations, with the same length and GC distribution as the positives. As Random Forest implementation we used the scikit-learn Python package [67] 151 decision trees were used for each classifier. Changing the number of trees can be indicative of the stabilisation of the cross-validation performance (S1 Fig). The parameter max_features (responsible for number of features to consider when looking for the best split) was set to sqrt(number of features). To calculate the feature importance we used the Gini impurity criterion averaged across trees, using the whole training data, again with the implementation from scikit-learn library [67].

### Comparison with existing CRM prediction methods

The performance of the RF classifiers was compared with simple PWM matching (M0) and with another supervised machine learning methods, namely kmerSVM (Mk) [29] and gapped kmerSVM (Mgk) [51]. The performance of the Mk, Mgk, M1, M2, M3 models where evaluated in 5-fold cross-validation. To evaluate performance of the M0 we obtained AuROC and AuPR curves varying the motif score threshold. For M0 we used as PWM matching tool MotifLocator [68] with default parameters except option -t was set to zero. For each TF we selected the PWM that was most enriched PWM in the training set. As a background model we used a first order Markov model with nucleotide transition probabilities estimated using human genome (hg19) sequence.

### Genome-wide scoring with RF classifiers

Genome-wide predictions were performed by segmenting the genome in overlapping sliding windows. The size of the window is chosen specifically for each TF as the average length of the regions used for training, and the overlapping segment between windows is equal to 200 bp.

### Scoring of simulated nucleotide substitutions

We selected a set of 752 known cancer drivers from different sources (MSigDB, TCGA, COSMIC). In the regions 900 bp upstream of these genes we replaced every nucleotide to each possible variant and scored with M1 models; PRIME score was calculated (difference between M1 classification score in mutant versus reference sequenc) to estimate contribution of the location and type of nucleotide substitution on the CRM score.

### Scoring of SNVs and insertions

The sequence around each mutation was scored with M1 models. Several sliding windows around each mutation were taken into account using a shift equal to 10% of the region. For each mutation the window with the maximum score of the classifier is taken into account.

### Conservation analysis

Bigwig file with phastCons scores [69] based on alignment of 46 placental mammal species was downloaded from UCSC Genome Browser. We used a custom Python script and bigWigToWig [70] tool to calculated the score for each position.

### Overlap with ChromHMM predictions

All chromatin states identified across nine human cell lines (HSMM, GM12878, HUVEC, H1-hESC, K562, HepG2, NHEK, HMEC, NHLF) using ChromHMM were downloaded from the UCSC browser [71] and combined into one dataset. We calculated the enrichment of positively predicted functional TF binding sites in different chromatin states using the GAT tool [72]. Only values where enrichment or depletion is significant (pvalue<0.05) are taken into account.

### TCGA breast cancer samples

From VCF files provided by TCGA consortium we selected non-coding somatic mutations (SNVs and insertions passed filtering criteria) falling in 500 bp regions around TSS. This yielded 51117 SNVs and 7323 insertions combined from 498 full-genome sequenced breast cancer samples. Z-scores of gene expression across samples were calculated using RPKM values (max value per gene) as derived from processed RNA-seq data for 768 breast cancer samples.

### The HeLa genome and epigenome

Processed full genome sequencing results of the HeLa cell line (CCL-2 and Kyoto cells) were downloaded as VCF files. Only insertions located in +- 10 kb non-coding regions around TSS and identified in both studies [43,73] were selected for scoring (N = 13923) and all heterozygos HeLa falling in H3K27Ac data (N = 89451). For the HeLa H3K27Ac data we used broadPeak formatted data generated by ENCODE (on the HeLa-S3 cells) [12] from which signalValue was used for creating z-scores as follows. Candidate regulatory regions (that we defined before [28]) were scored by a large collection of 109 H3K27Ac ChIP-seq data across different cell types including HeLa (46 datasets from Blueprint project [74,75], 23 from ENCODE [12], 3 from DEEP [76], 33 from McGill EMC (http://epigenomesportal.ca) and 4 in-house generated datasets). The acetylation score was multiplied by the fraction of the peak length that overlaps with the candidate regulatory region. If more than one peak overlaps with the same regulatory region then the average value was used. Finally, each regulatory region had a score for all the 109 acetylation datasets and z-scores were computed across all the samples.

### Availability of software code

Python scripts are available at https://github.com/aertslab/primescore.

## Supporting Information

S1 Fig Example ROC curves. ROC curves for two example models, BATF and ATF2, showing the increasing performance of M1 compared to M0, and of M3 compared to M1.
(TIFF)

S2 Fig Random Forest stabilization. AuPR of the M1 models depending on the number or trees. Varying the number of trees in the forest demonstrates stabilization of the classifier performance (AuPR) for the majority of the models. For some models (POU5F1, NANOG) fluctuations are higher due to the low number of training samples.
(TIFF)

S3 Fig Comparison of machine learning methods using the AuPR. Heatmap with AuPR scores for Logistic regression (LR), SVM and Random Forest (RF) classifiers. We compared RF classifiers with two other supervised machine learning methods using the same data and features. For all models the RF classifier outperforms other learning algorithms. Also, increasing complexity of the also yields higher performance. (A) AuPR values for M1 models using motifs only; (B) AuPR values for M2 models, using tracks only; (C) AuPR values for M3 models using both motifs and tracks.
(TIFF)

S4 Fig Comparison of machine learning methods using the ROC. AuROC for LR, SVM and Random Forest (RF) classifiers. AuROC for LR and SVM are lower than for RF considering the same training data and features. (A) AuROC values for M1 models using motifs only; (B) AuROC values for M2 models, using tracks only; (C) AuROC values for M3 models using both motifs and tracks.
(TIFF)

S5 Fig Performance versus number of training samples. Performance (AuPR) of the M1 models in cross-validation does not depend on the number of training CRMs. For the three models (ESR1, MYC and YY1) having more than 2000 training CRMs performance is relatively high but not bigger then for some models with less then 200 samples.
(TIFF)

S6 Fig Performance versus PWM information content. AuPR vs information content of the PWMs of M1 model. A) There is no clear dependence between the average information content of the PWMs used by M1 and AuPR achieved in cross-validation. B) Furthermore, the most informative PWMs do not lead to higher classifier performance.
(TIFF)

S7 Fig Feature importance for 45 Random Forest models. Heatmap showing the summed Gini importance averaged across tries for each group of features (M3 model). The higher values mean larger contribution of the attributes to the classification decision.
(TIFF)

S8 Fig Comparison of genome-wide scoring results between models. Correlation of the TF ChIP-seq peak enrichment scores for genome wide predictions obtained with Mk, M1, M3 models. Random forest models (M1 and M3) utilizing various set of features show high agreement with each other (r = 0.876) and both models are less correlated with the TF ChIP-seq peak enrichment of predictions obtained with Mk. This demonstrates that for the same TFs both RF classifiers (M1 and M3) have similar enrichment of the corresponding ChIP-seq peaks in the newly predicted CRMs. Diagonal shows density profile of the enrichment scores for each of the 45 models from M1, M3 and Mk.
(TIFF)

S9 Fig Enrichment of newly predicted functional CRMs in various chromatin states. For all genome-wide predicted (M1) functional CRMs (excluding training regions) with score above 0.5 we calculated the enrichment of overlap with chromatin states obtained with chromHMM across 9 cell lines. Values on the heatmap show significant (p-value<0.05) log2 fold ratio of the observed overlap against expected by chance. Non significant values were set to zero.
(TIFF)

S10 Fig Comparison of PRIME scores with sequence constraint inside and outside real binding sites. High PRIME score nucleotides overlapping with true binding sites are under higher constraint compared to nucleotides outside of the ChIP-seq peaks. Nevertheless, high-scoring mutations outside experimentally identified TF binding sites are enriched for high phastCons scores.
(TIFF)

S11 Fig DNAseI-seq profile around high-scoring (>0.3) nucleotides. Simulated substitutions (center of x-axis) with high PRIME scores are located in more accessible regions than substitutions with low scores (<0.01) suggesting their potential involvement in CRM function. The DNAseI-seq data shown here was obtained for the A549 cell line by the ENCODE consortium.
(TIFF)

S12 Fig Cancer mutations with high PRIME scores are under constraint. All scored somatic mutations from AML (N = 50), melanoma (N = 25) and breast cancer (N = 21) samples are pooled. With increasing PRIME score we observe a trend towards an increase of the average nucleotide conservation measured by the phastCons score.
(TIFF)

S13 Fig Enrichment of high scoring mutations in chromHMM states. Non-coding mutations with high PRIME scores show much stronger enrichment in regulatory active chromatin states (promoters and enhancers) compared to all mutations in the group.
(TIFF)

S14 Fig Specificity of the M1 models for scoring non-coding somatic mutations. Non-coding somatic mutations found in breast cancer samples with absolute PRIME score>0.4 (N = 911) where checked for specificity with M1 and M0 models. Simulated possible nucleotide substitutions in the window around mutations where scored and ranked. The plot demonstrates the rank recovery of the true non-coding mutations ranked according to PRIME scores (M1) and delta PWM scores (M0), demonstrating greater specificity of the Random Forest models comparing to PWMs.
(TIFF)

S15 Fig Specificity of MYB gain of function PRIME scores. We inserted exactly the same sequence as found in Jurkat, MOLT-3 and patient samples (P6, P8) at 100 randomly chosen genomic loci having the same 3bp flanking nucleotides. The PRIME score strongly depends on the surrounding sequence context and for example, the Jurkat insertion generates a PRIME score equal or higher than 0.32 (the observed PRIME in the TAL1 enhancer) in only 10/100 locations.
(TIFF)

S16 Fig Correlation of PRIME scores with gene expression changes. Violin and boxplots show an association of changes in gene expression with predicted impact of promoter mutations. The median absolute z-score values of gene expression increase with increasing PRIME score. Also, the expression changes in the low PRIME group (PRIME below 0.03) are less comparing to high scoring groups.
(TIFF)

S17 Fig Association of PRIME scores with H3K27Ac in HeLa. A) Scatter plot of PRIME scores (45 M1 models) for insertions in the HeLa cell line versus z-scores of H3K27ac peak scores. The most upper left point indicates an insertion near CDH10 with high PRIME score (-0.295 for POU5F1 and -0.274 for NANOG), which also has a high H3K27ac z-score. B) Illustration of CDH10 regulatory insertion, with the H3K27Ac signal around this mutation found exclusively in the HeLa cell line, not in other ENCODE cell lines. The red box indicates the position of the insertion.
(TIFF)

S18 Fig Cross-validation results of the gkm-SVM method. Area under precision-recall (AuPR) and receiver operating characteristic (AuROC) curves for gapped kmer-SVM (Mgk) compared to M1 models, estimated by 5-fold cross-validation. Both methods demonstrate comparable results with slight outperformance on average for M1.
(TIFF)

S19 Fig Comparison of genome-wide scoring results between gkm-SVM and M1. After genome-wide CRM scoring, removing the training CRMs, evaluating the enrichment of ChIP-seq peaks of the corresponding TF, and the enrichment of motifs of the corresponding TF, within the top 1000 newly predicted CRMs. Enrichment is represented as a Normalized Enrichment Score (NES) calculated by i-cisTarget. A) Significant enrichment of ChIP-seq peaks (orange is NES>2.5) for 31/45 M1 models, compared to 12/45 of the Mgk models. B) The motif of the respective TF is also enriched in the top 1000 newly predicted functional CRMs, for those in orange (NES>2.5).
(TIFF)

S20 Fig Comparison of the specificity of M1 and M0 sum models. For the “M0 sum” model we summed the maximal motif scores (using the same PWMs as for M1 model) found in the 900bp regions upstream of MTM1 gene. Possible nucleotide substitutions demonstrate that M1 PRIME score are more specific and most scoring nucleotides are within the ChIPed region, which is not the case for M0 sum model.
(TIFF)

S21 Fig Comparison with deltaSVM on hepG2 enhancers. M1 and deltaSVM models (trained on the same sequences for NFE2L2 and HNF4A TFs) show association of the delta scores (predicted impact, x-axis) with reporter expression changes (y-axis). Both methods demonstrate comparable performance.
(TIFF)

S22 Fig Comparison with deltaSVM regarding specificity. M1 and deltaSVM models for IRF1, SPI1, E2F4 and FOSL1 models where applied to predict the impact of simulated nucleotide substitutions. Both methods demonstrate good agreement with each other identifying the highest scoring nucleotides within the ChIP’ped regions for IRF1 and SPI1 but E2F4 and FOSL1 models are more specific for the Random Forest M1 model than for deltaSVM.
(TIFF)

S23 Fig Cross-validation with i-cisTarget feature filtering per fold. The performance of the M1 models using features selected on the entire dataset is comparable (r = 0.978) with models utilizing features identified only using training subset of the data and applied to the test data not participated in the selection.
(TIFF)

S1 Table Number of training CRMs for each TF. (XLSX)

S2 Table AuPR and AuROC for M0, Mk, M1, M2, M3. (XLSX)

S3 Table SNVs in TCGA breast cancer genomes, in promoters of cancer-related genes and in 20kB around TSS of breast-cancer specific TFs, with significant PRIME (z-score>9.65). (XLSX)

S4 Table Small insertions in TCGA breast cancer genomes, in promoters of cancer-related genes, and in 10kb upstream of breast-cancer specific TFs, with high absolute PRIME score (z-score>14.00). (XLSX)

S5 Table Number of used features for each model. (XLSX)

S6 Table PRIME score threshold for each model based on the insertion in the TAL1 enhancer in the MOLT-3 cell line. (XLSX)
