# Supplementary figures and images for: Identification of High-Impact cis-Regulatory Mutations Using Transcription Factor Specific Random Forest Models
Source: PLoS Comput Biol. 2015 Nov 12;11(11):e1004590. doi: 10.1371/journal.pcbi.1004590 (PMC4642938; doi:10.1371/journal.pcbi.1004590)

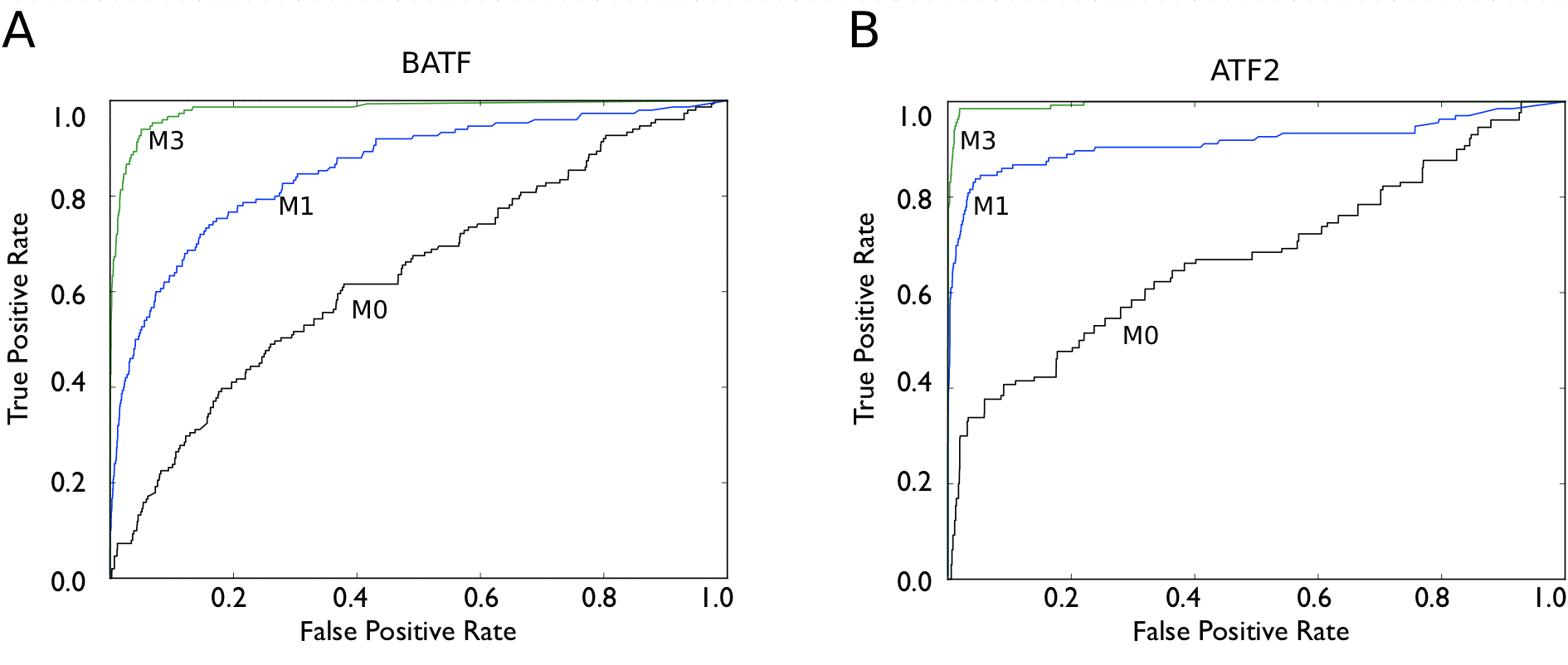

Supplement: S1 Fig — ROC curves for two example models, BATF and ATF2, showing the increasing performance of M1 compared to M0, and of M3 compared to M1. (TIFF) [file pcbi.1004590.s001.tiff]

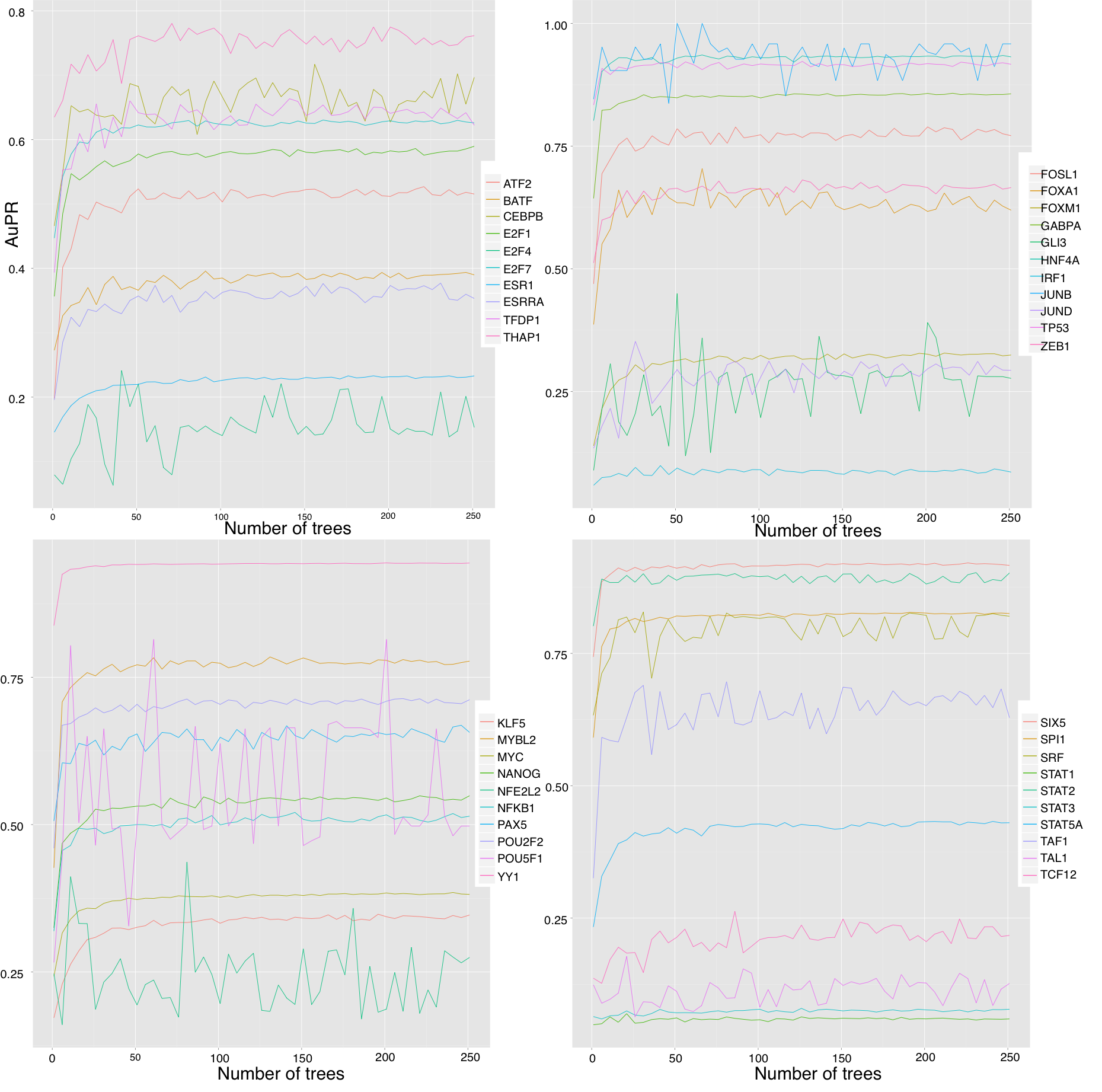

Supplement: S2 Fig — AuPR of the M1 models depending on the number or trees. Varying the number of trees in the forest demonstrates stabilization of the classifier performance (AuPR) for the majority of the models. For some models (POU5F1, NANOG) fluctuations are higher due to the low number of training samples. (TIFF) [file pcbi.1004590.s002.tiff]

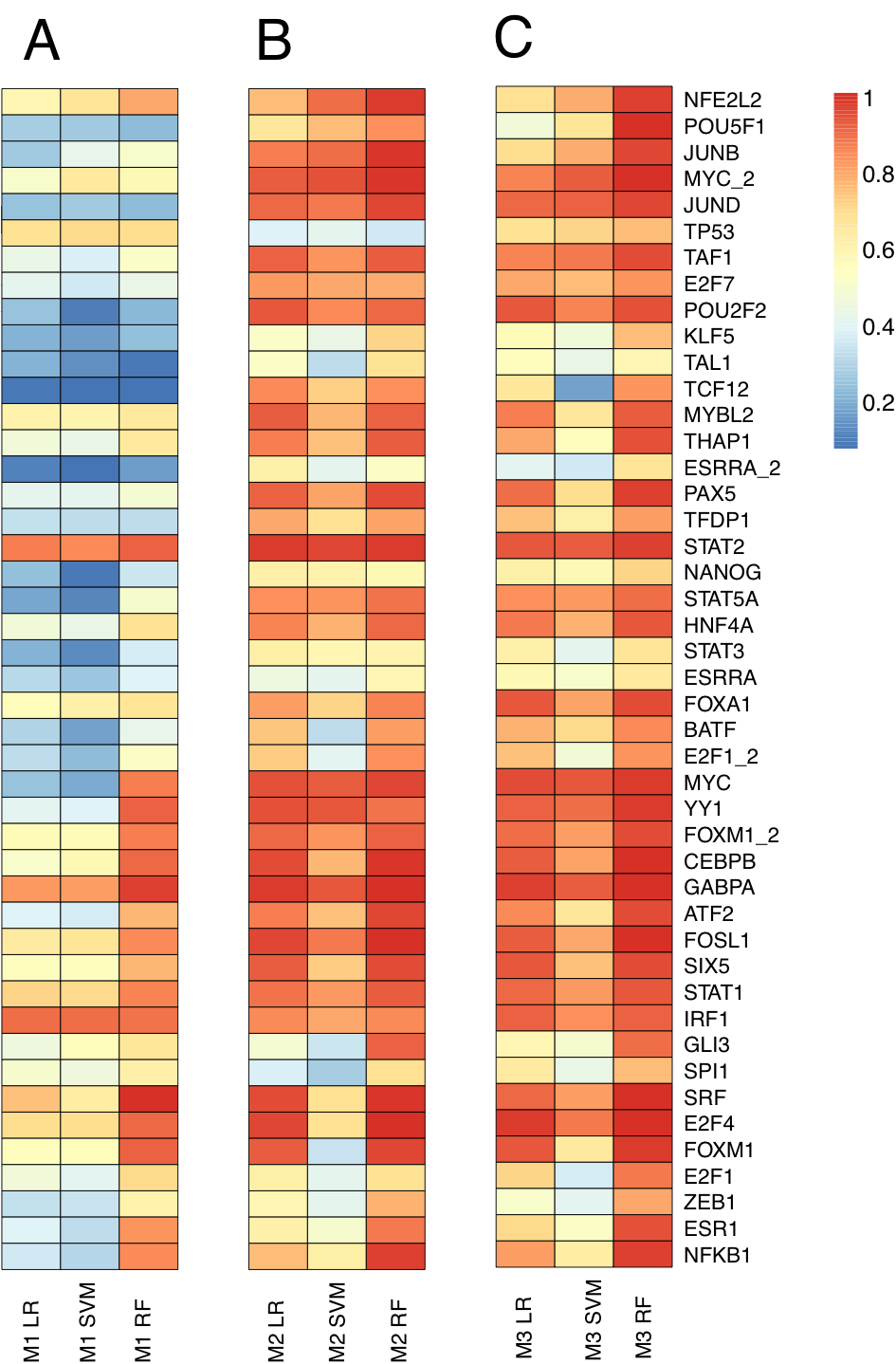

Supplement: S3 Fig — Heatmap with AuPR scores for Logistic regression (LR), SVM and Random Forest (RF) classifiers. We compared RF classifiers with two other supervised machine learning methods using the same data and features. For all models the RF classifier outperforms other learning algorithms. Also, increasing complexity of the also yields higher performance. (A) AuPR values for M1 models using motifs only; (B) AuPR values for M2 models, using tracks only; (C) AuPR values for M3 models using both motifs and tracks. (TIFF) [file pcbi.1004590.s003.tiff]

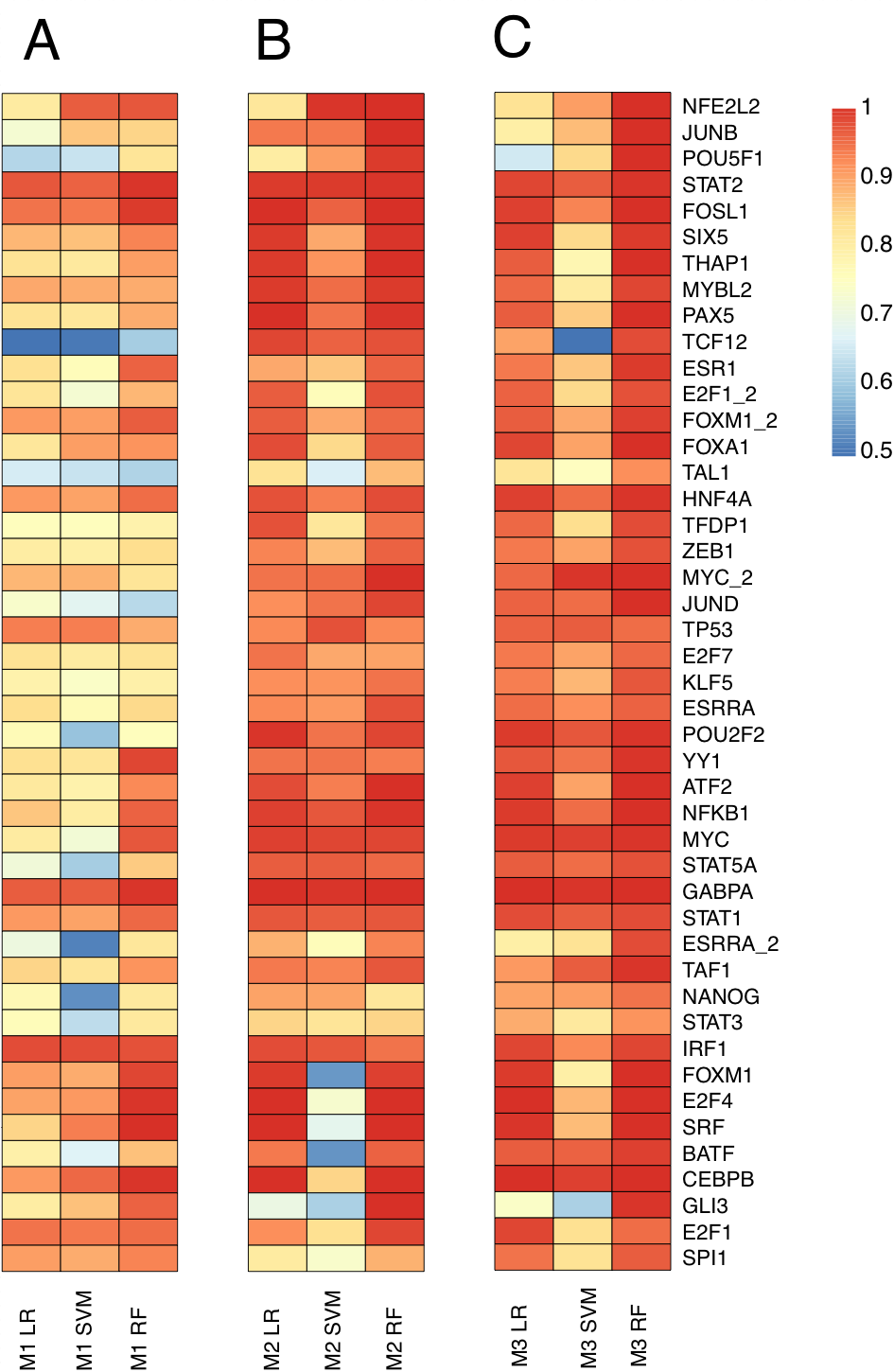

Supplement: S4 Fig — AuROC for LR, SVM and Random Forest (RF) classifiers. AuROC for LR and SVM are lower than for RF considering the same training data and features. (A) AuROC values for M1 models using motifs only; (B) AuROC values for M2 models, using tracks only; (C) AuROC values for M3 models using both motifs and tracks. (TIFF) [file pcbi.1004590.s004.tiff]

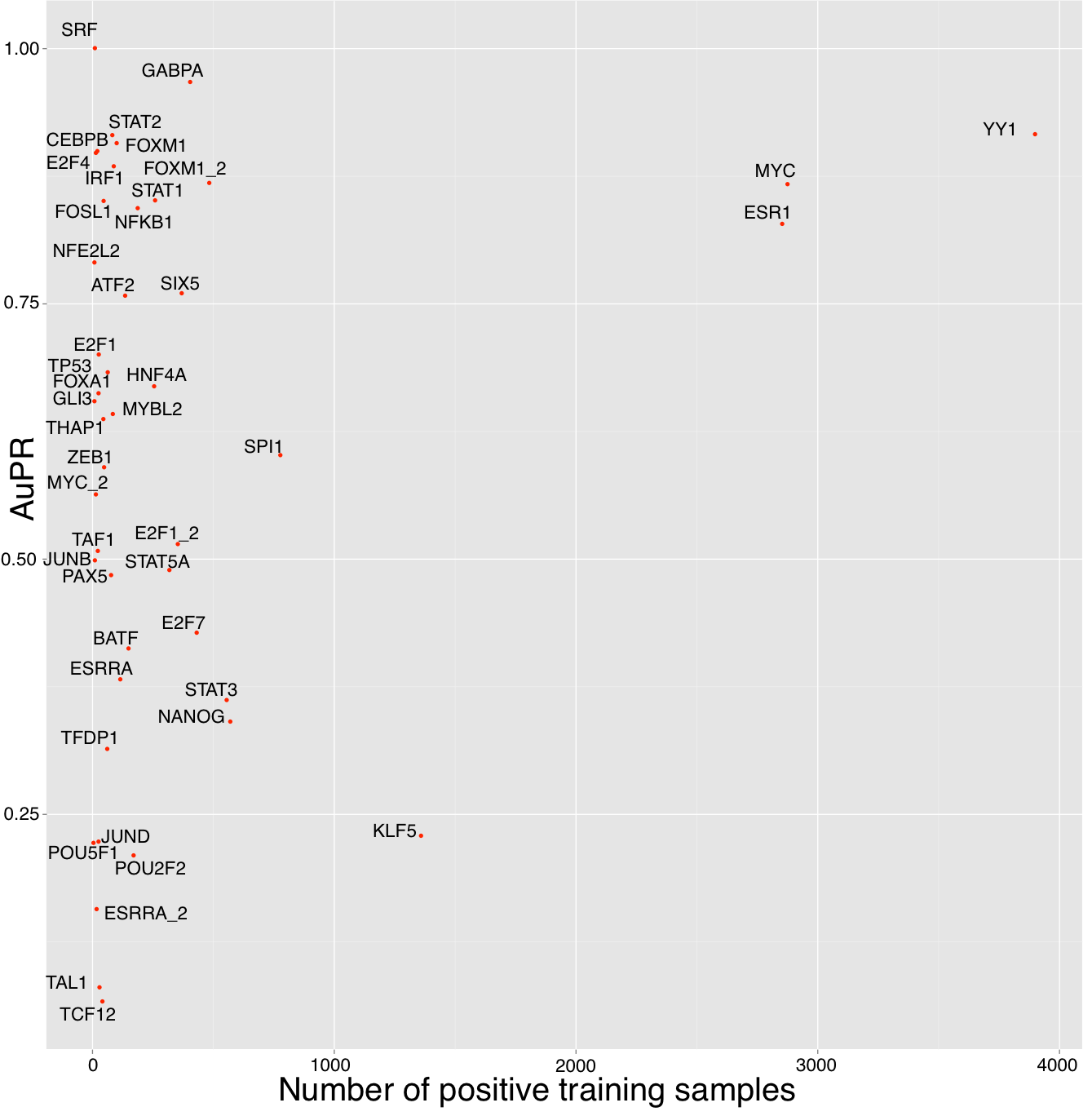

Supplement: S5 Fig — Performance (AuPR) of the M1 models in cross-validation does not depend on the number of training CRMs. For the three models (ESR1, MYC and YY1) having more than 2000 training CRMs performance is relatively high but not bigger then for some models with less then 200 samples. (TIFF) [file pcbi.1004590.s005.tiff]

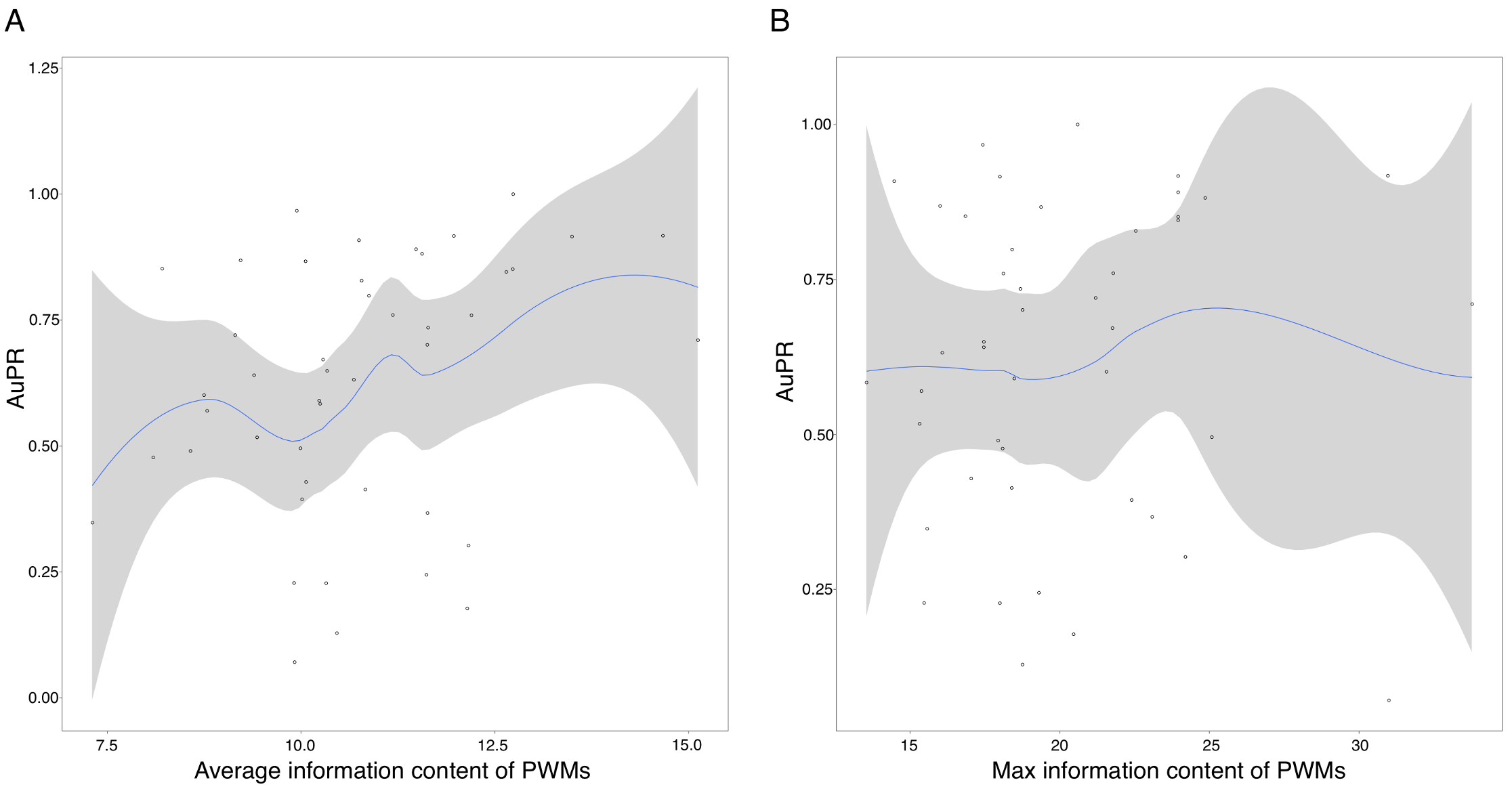

Supplement: S6 Fig — AuPR vs information content of the PWMs of M1 model. A) There is no clear dependence between the average information content of the PWMs used by M1 and AuPR achieved in cross-validation. B) Furthermore, the most informative PWMs do not lead to higher classifier performance. (TIFF) [file pcbi.1004590.s006.tiff]

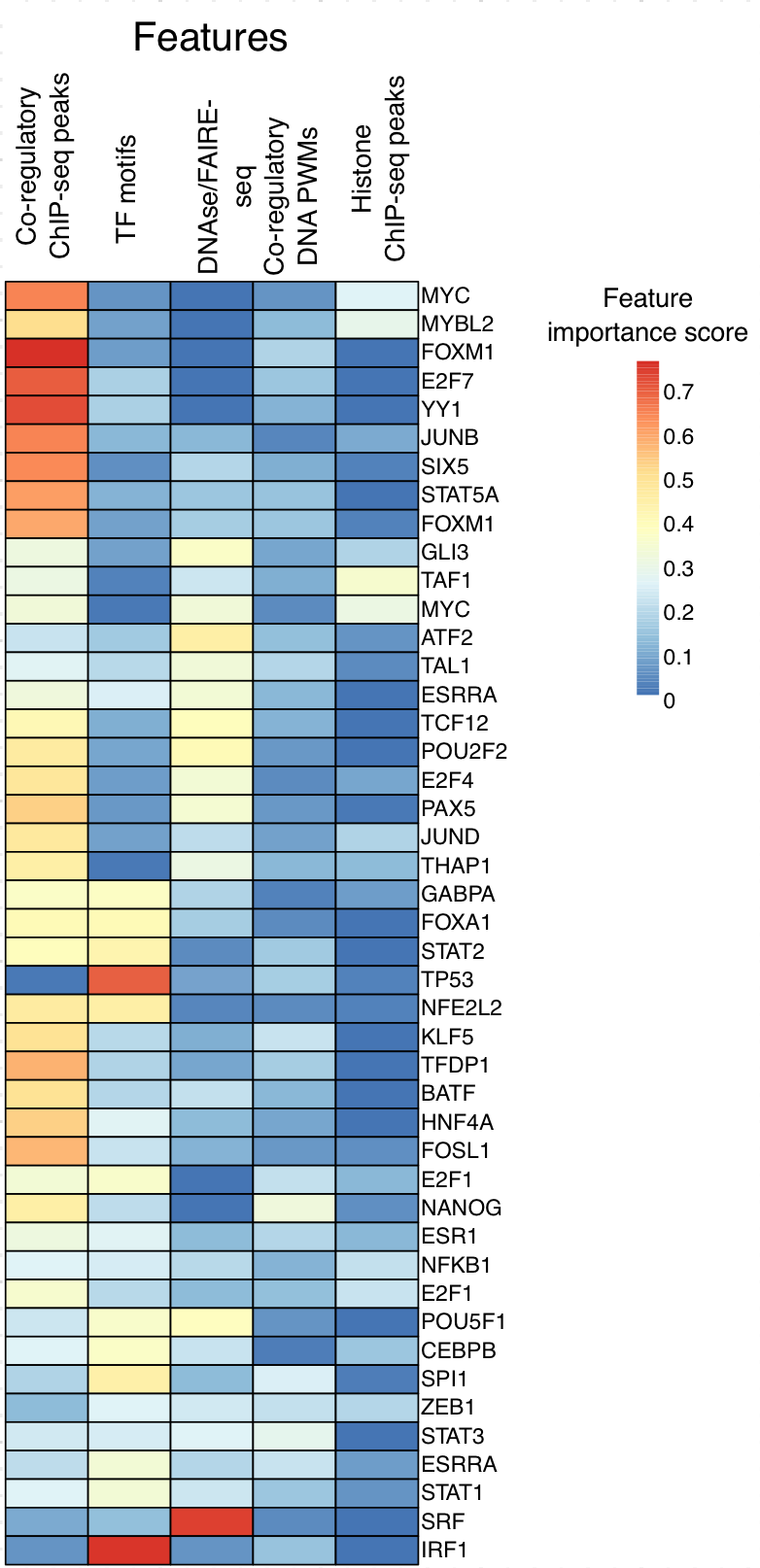

Supplement: S7 Fig — Heatmap showing the summed Gini importance averaged across tries for each group of features (M3 model). The higher values mean larger contribution of the attributes to the classification decision. (TIFF) [file pcbi.1004590.s007.tiff]

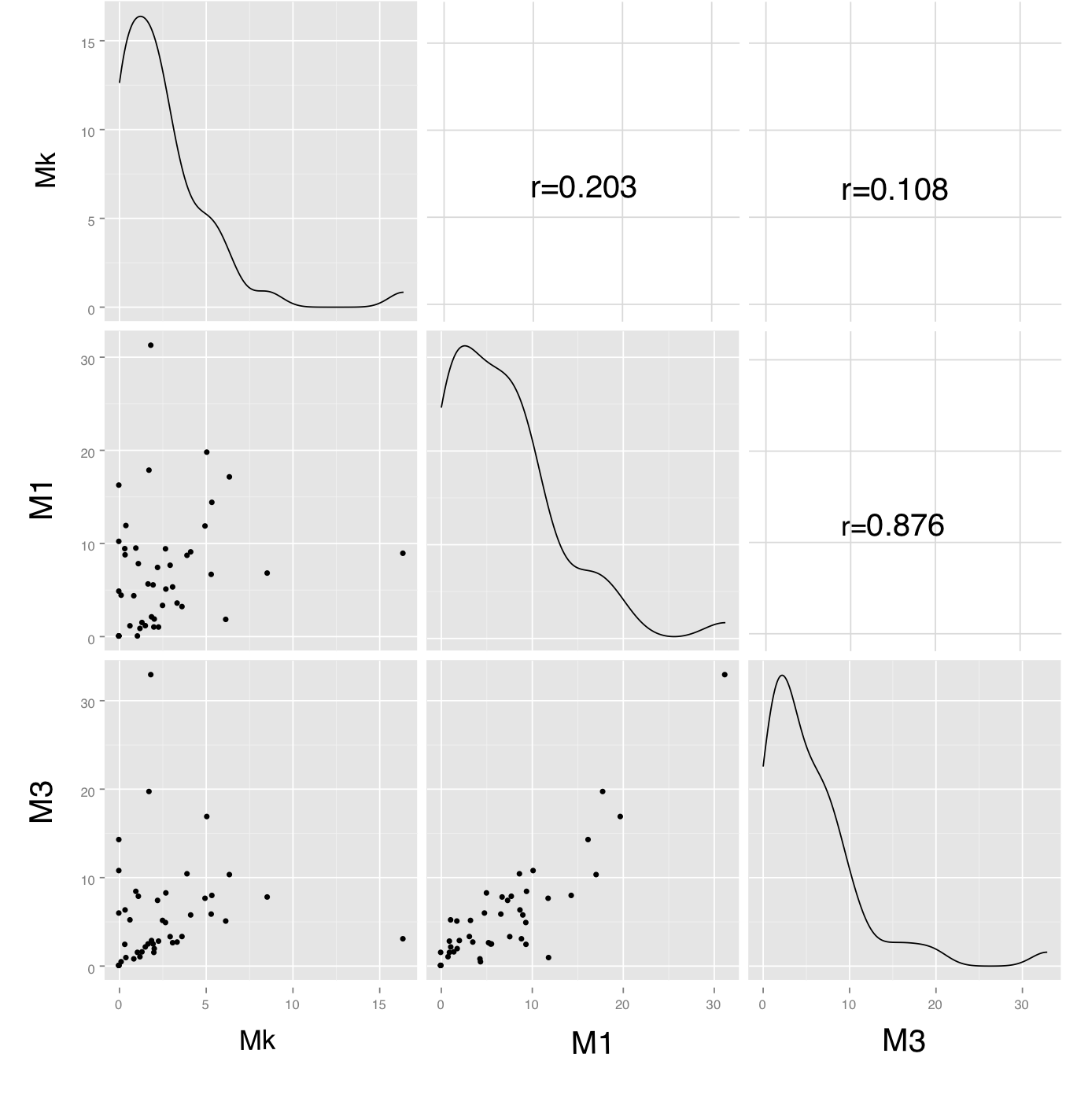

Supplement: S8 Fig — Correlation of the TF ChIP-seq peak enrichment scores for genome wide predictions obtained with Mk, M1, M3 models. Random forest models (M1 and M3) utilizing various set of features show high agreement with each other (r = 0.876) and both models are less correlated with the TF ChIP-seq peak enrichment of predictions obtained with Mk. This demonstrates that for the same TFs both RF classifiers (M1 and M3) have similar enrichment of the corresponding ChIP-seq peaks in the newly predicted CRMs. Diagonal shows density profile of the enrichment scores for each of the 45 models from M1, M3 and Mk. (TIFF) [file pcbi.1004590.s008.tiff]

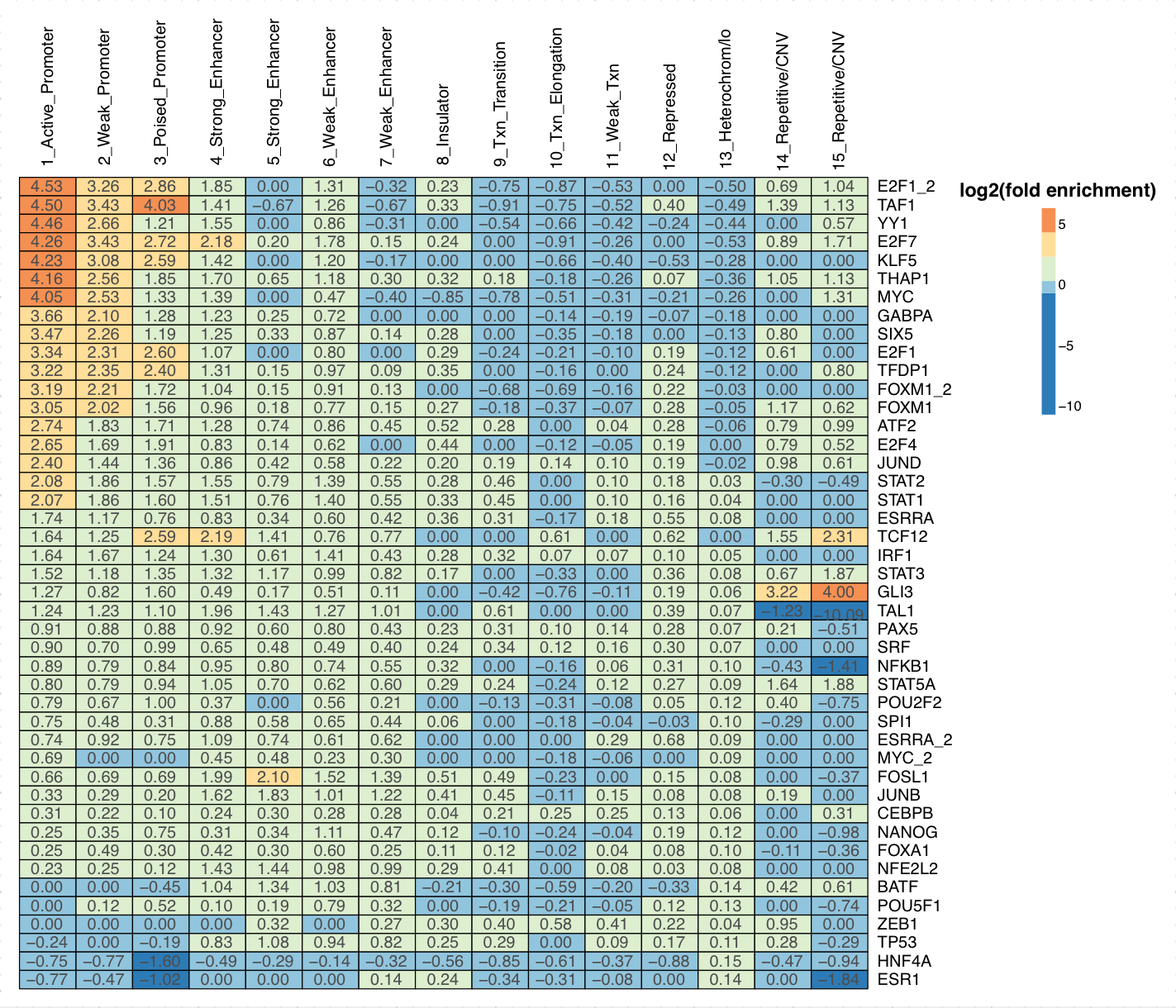

Supplement: S9 Fig — For all genome-wide predicted (M1) functional CRMs (excluding training regions) with score above 0.5 we calculated the enrichment of overlap with chromatin states obtained with chromHMM across 9 cell lines. Values on the heatmap show significant (p-value<0.05) log2 fold ratio of the observed overlap against expected by chance. Non significant values were set to zero. (TIFF) [file pcbi.1004590.s009.tiff]

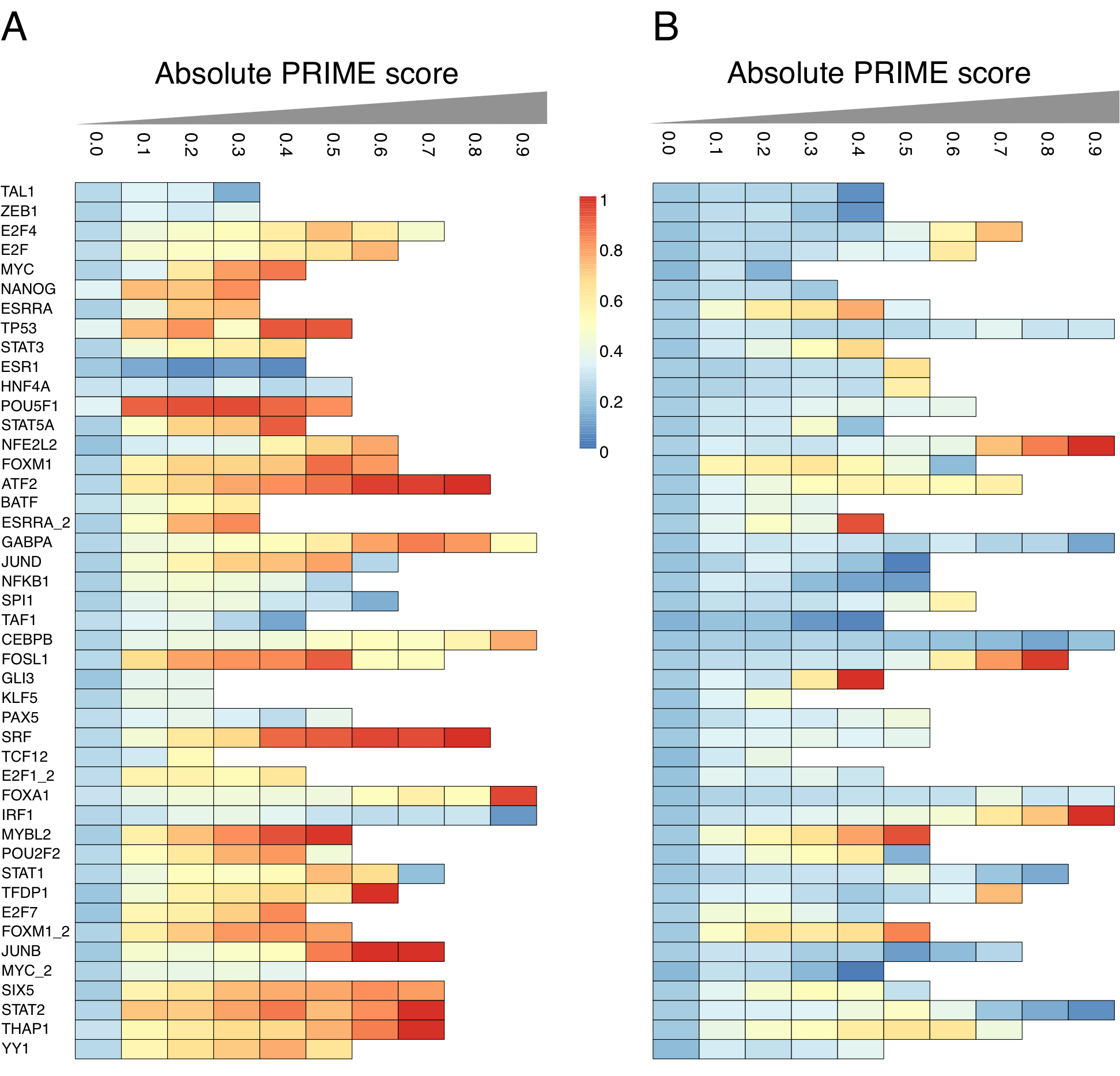

Supplement: S10 Fig — High PRIME score nucleotides overlapping with true binding sites are under higher constraint compared to nucleotides outside of the ChIP-seq peaks. Nevertheless, high-scoring mutations outside experimentally identified TF binding sites are enriched for high phastCons scores. (TIFF) [file pcbi.1004590.s010.tiff]

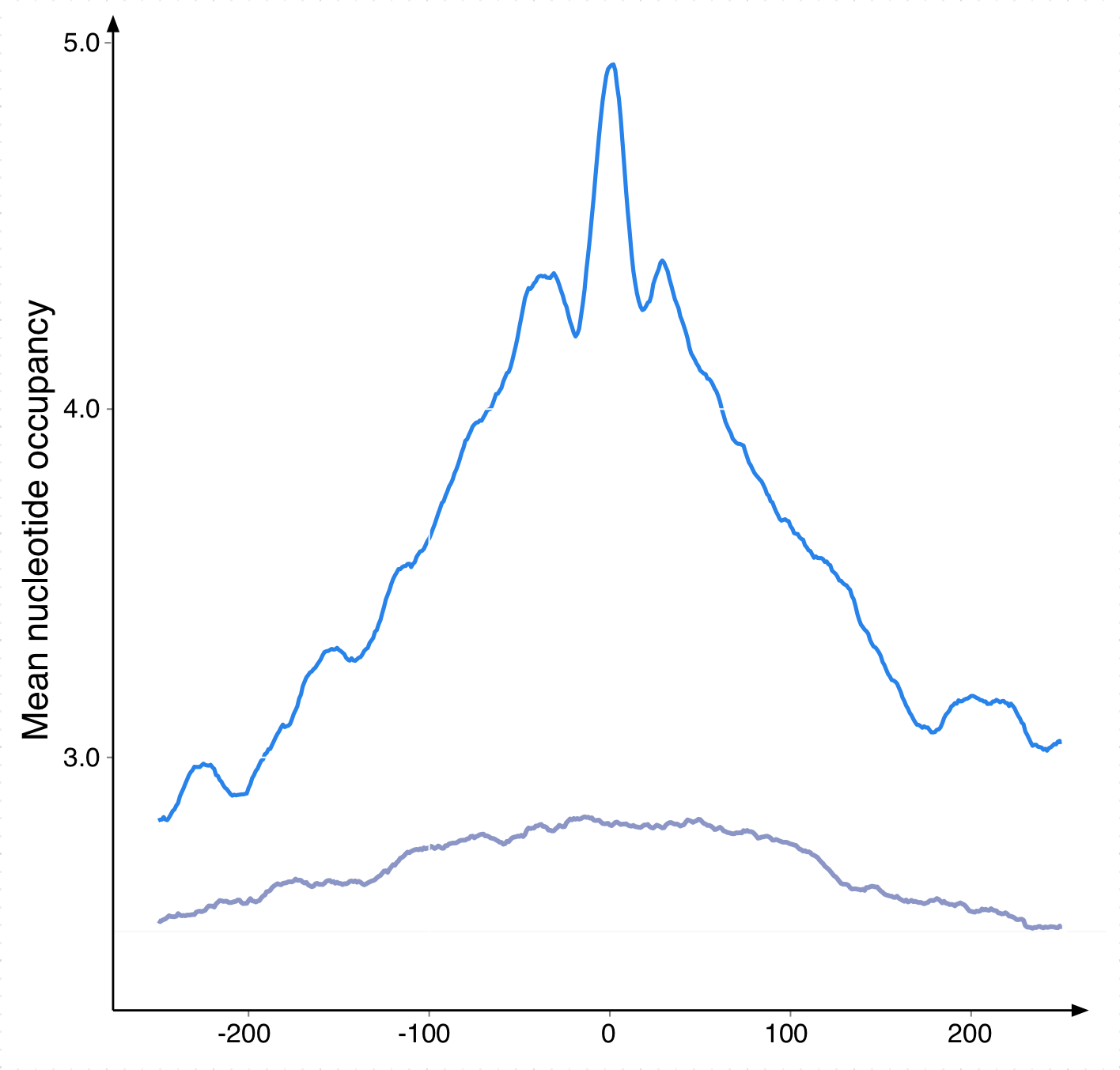

Supplement: S11 Fig — Simulated substitutions (center of x-axis) with high PRIME scores are located in more accessible regions than substitutions with low scores (<0.01) suggesting their potential involvement in CRM function. The DNAseI-seq data shown here was obtained for the A549 cell line by the ENCODE consortium. (TIFF) [file pcbi.1004590.s011.tiff]

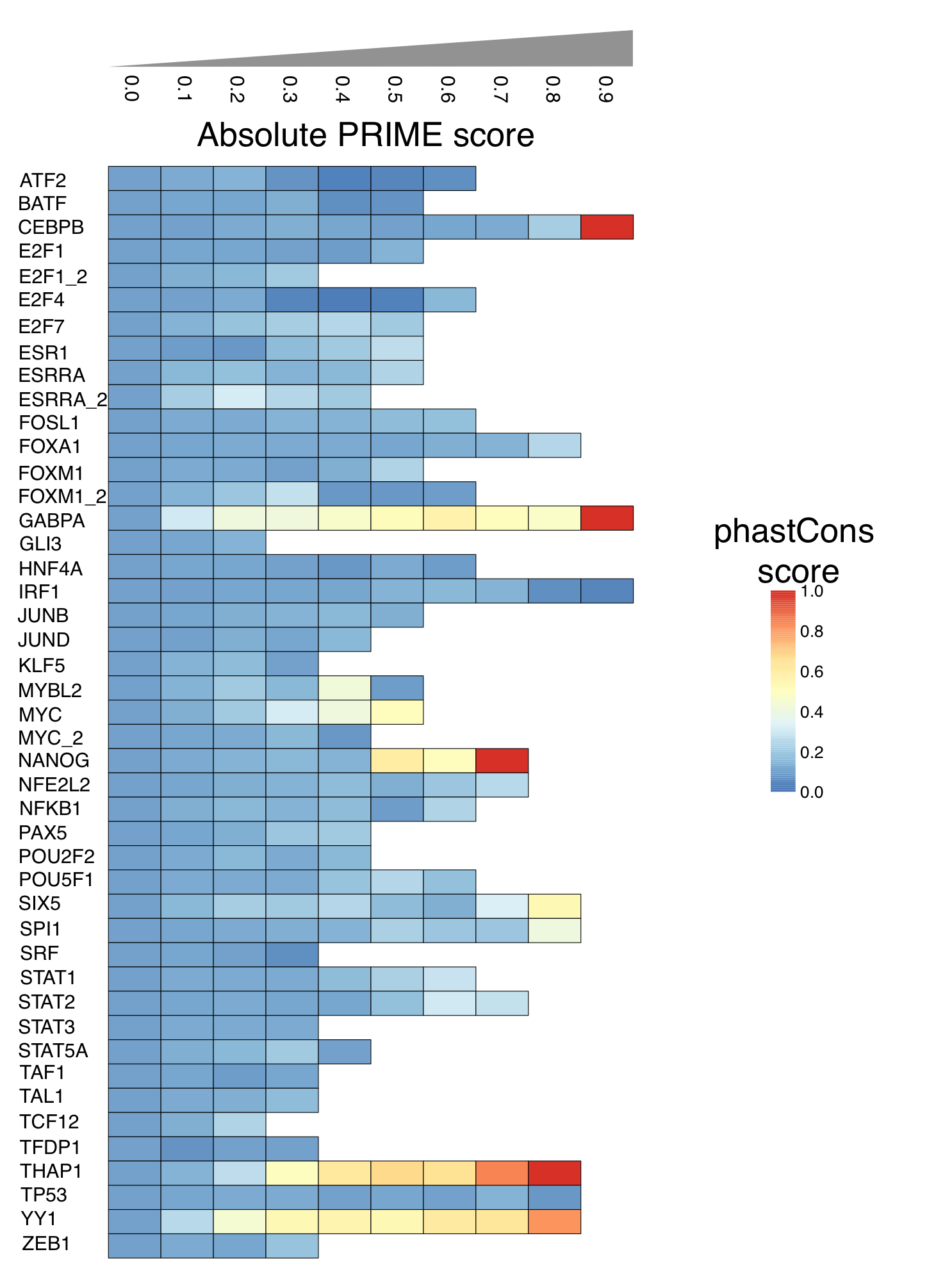

Supplement: S12 Fig — All scored somatic mutations from AML (N = 50), melanoma (N = 25) and breast cancer (N = 21) samples are pooled. With increasing PRIME score we observe a trend towards an increase of the average nucleotide conservation measured by the phastCons score. (TIFF) [file pcbi.1004590.s012.tiff]

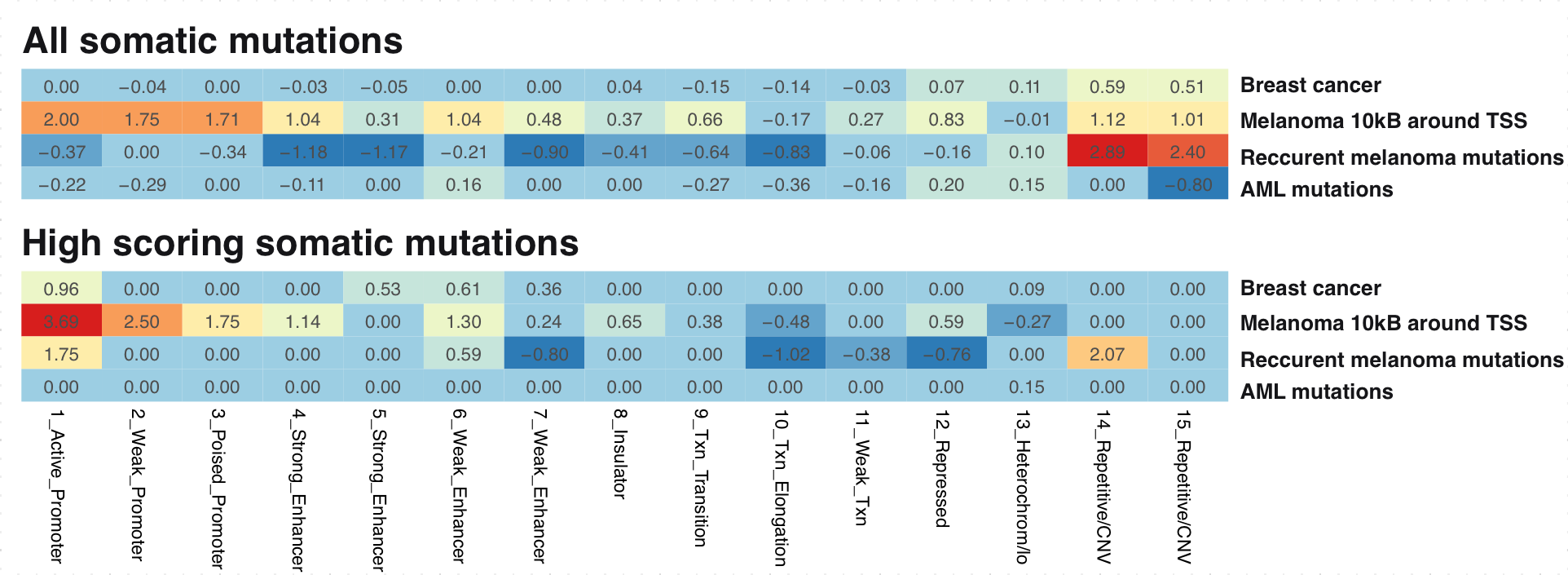

Supplement: S13 Fig — Non-coding mutations with high PRIME scores show much stronger enrichment in regulatory active chromatin states (promoters and enhancers) compared to all mutations in the group. (TIFF) [file pcbi.1004590.s013.tiff]

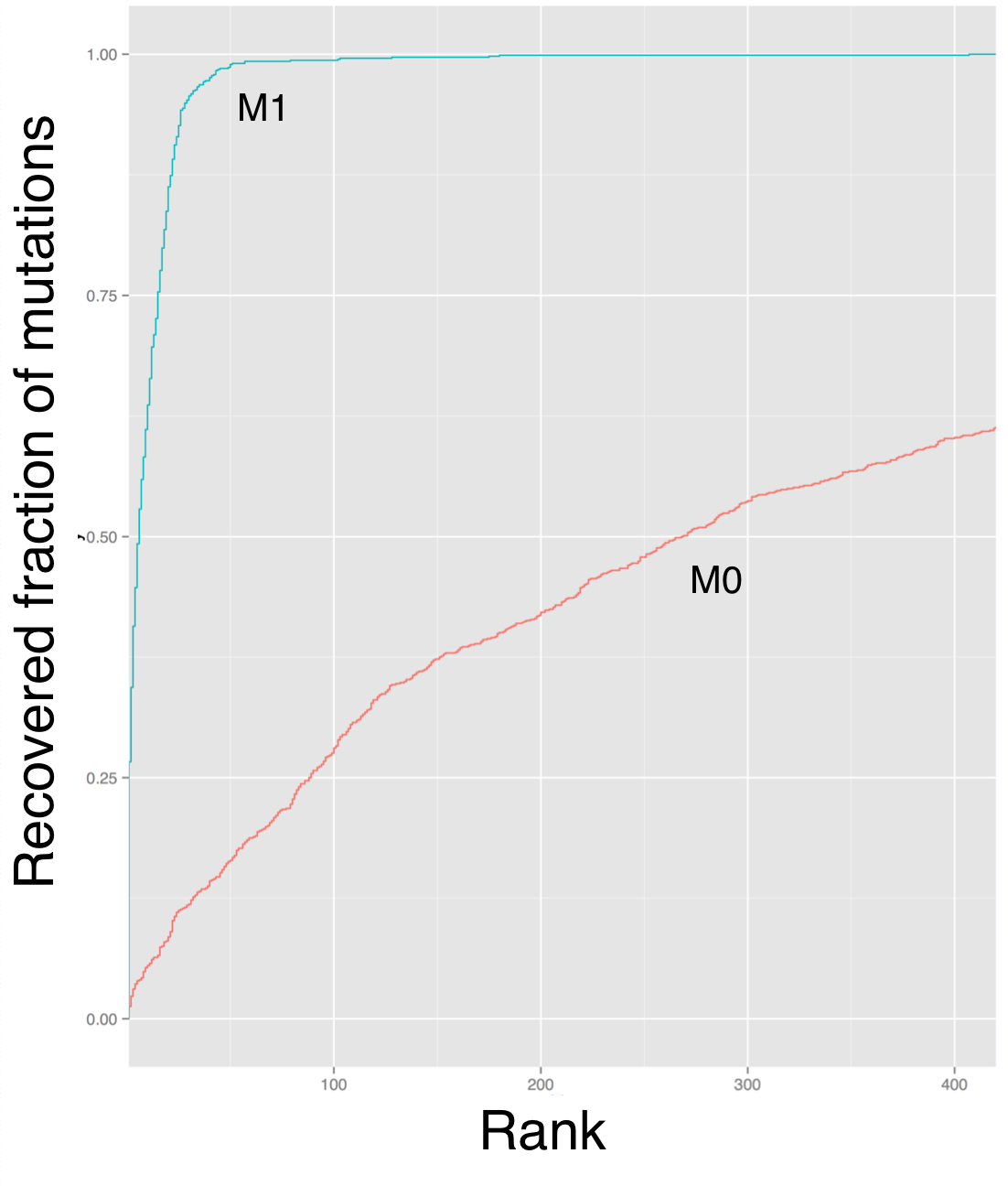

Supplement: S14 Fig — Non-coding somatic mutations found in breast cancer samples with absolute PRIME score>0.4 (N = 911) where checked for specificity with M1 and M0 models. Simulated possible nucleotide substitutions in the window around mutations where scored and ranked. The plot demonstrates the rank recovery of the true non-coding mutations ranked according to PRIME scores (M1) and delta PWM scores (M0), demonstrating greater specificity of the Random Forest models comparing to PWMs. (TIFF) [file pcbi.1004590.s014.tiff]

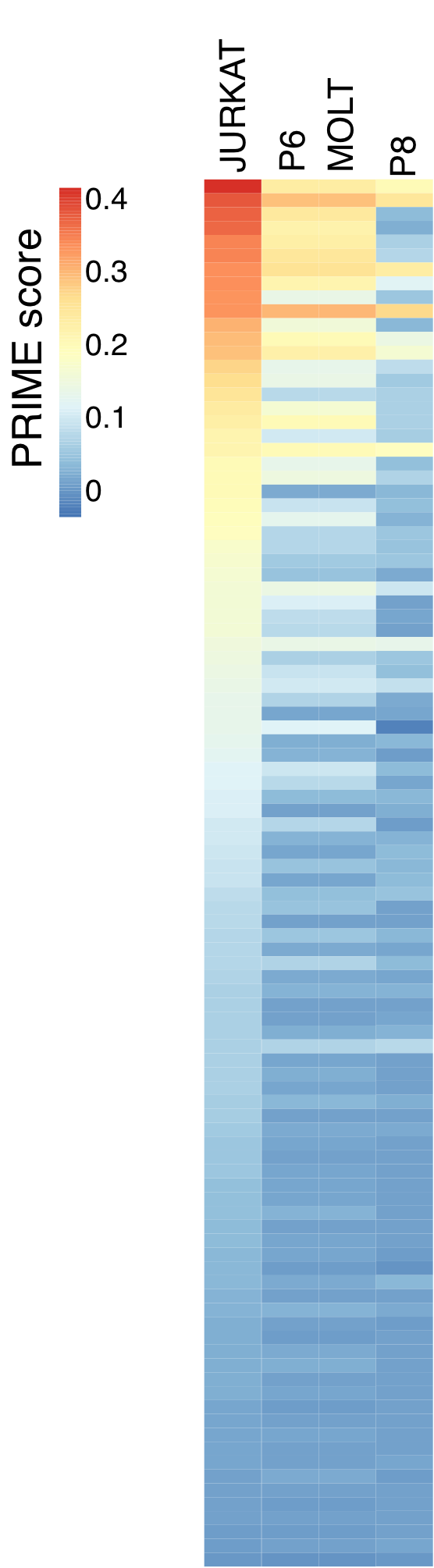

Supplement: S15 Fig — We inserted exactly the same sequence as found in Jurkat, MOLT-3 and patient samples (P6, P8) at 100 randomly chosen genomic loci having the same 3bp flanking nucleotides. The PRIME score strongly depends on the surrounding sequence context and for example, the Jurkat insertion generates a PRIME score equal or higher than 0.32 (the observed PRIME in the TAL1 enhancer) in only 10/100 locations. (TIFF) [file pcbi.1004590.s015.tiff]

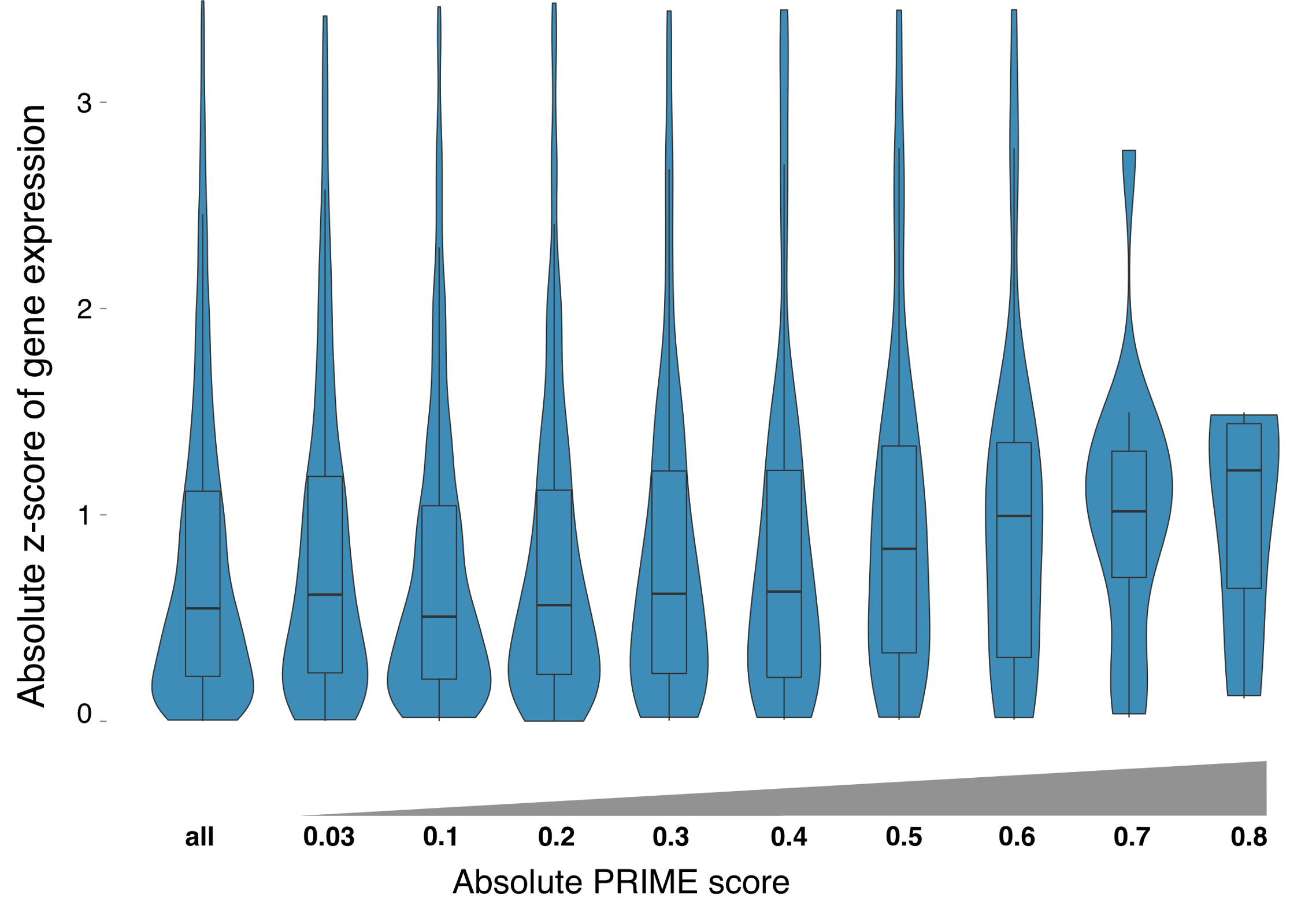

Supplement: S16 Fig — Violin and boxplots show an association of changes in gene expression with predicted impact of promoter mutations. The median absolute z-score values of gene expression increase with increasing PRIME score. Also, the expression changes in the low PRIME group (PRIME below 0.03) are less comparing to high scoring groups. (TIFF) [file pcbi.1004590.s016.tiff]

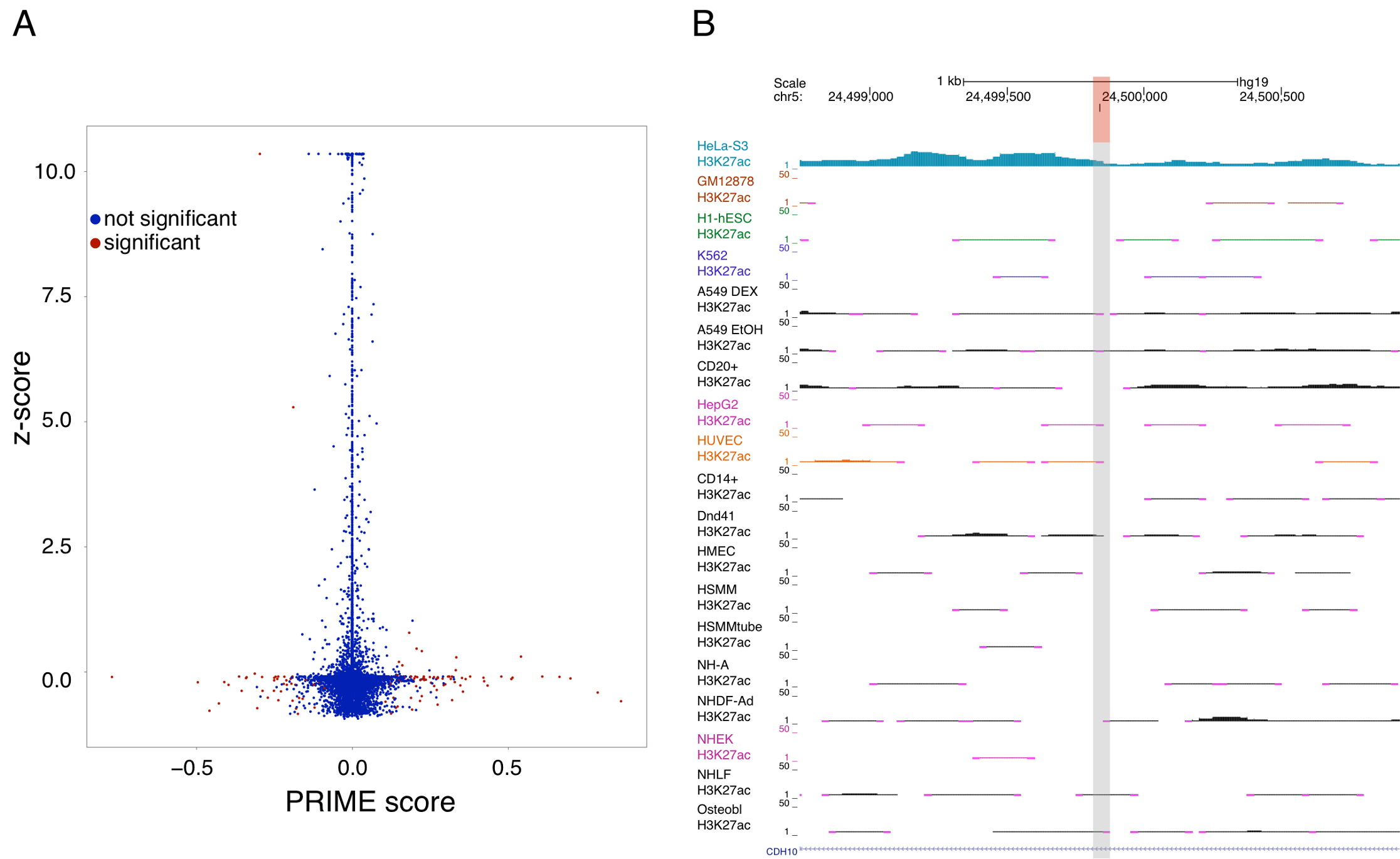

Supplement: S17 Fig — A) Scatter plot of PRIME scores (45 M1 models) for insertions in the HeLa cell line versus z-scores of H3K27ac peak scores. The most upper left point indicates an insertion near CDH10 with high PRIME score (-0.295 for POU5F1 and -0.274 for NANOG), which also has a high H3K27ac z-score. B) Illustration of CDH10 regulatory insertion, with the H3K27Ac signal around this mutation found exclusively in the HeLa cell line, not in other ENCODE cell lines. The red box indicates the position of the insertion. (TIFF) [file pcbi.1004590.s017.tiff]

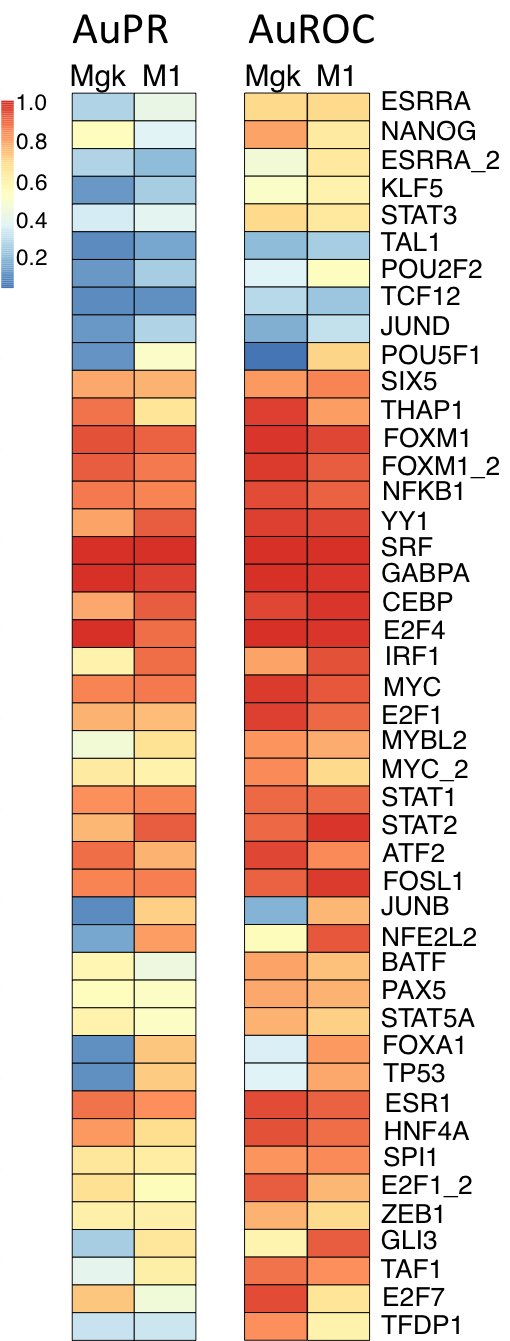

Supplement: S18 Fig — Area under precision-recall (AuPR) and receiver operating characteristic (AuROC) curves for gapped kmer-SVM (Mgk) compared to M1 models, estimated by 5-fold cross-validation. Both methods demonstrate comparable results with slight outperformance on average for M1. (TIFF) [file pcbi.1004590.s018.tiff]

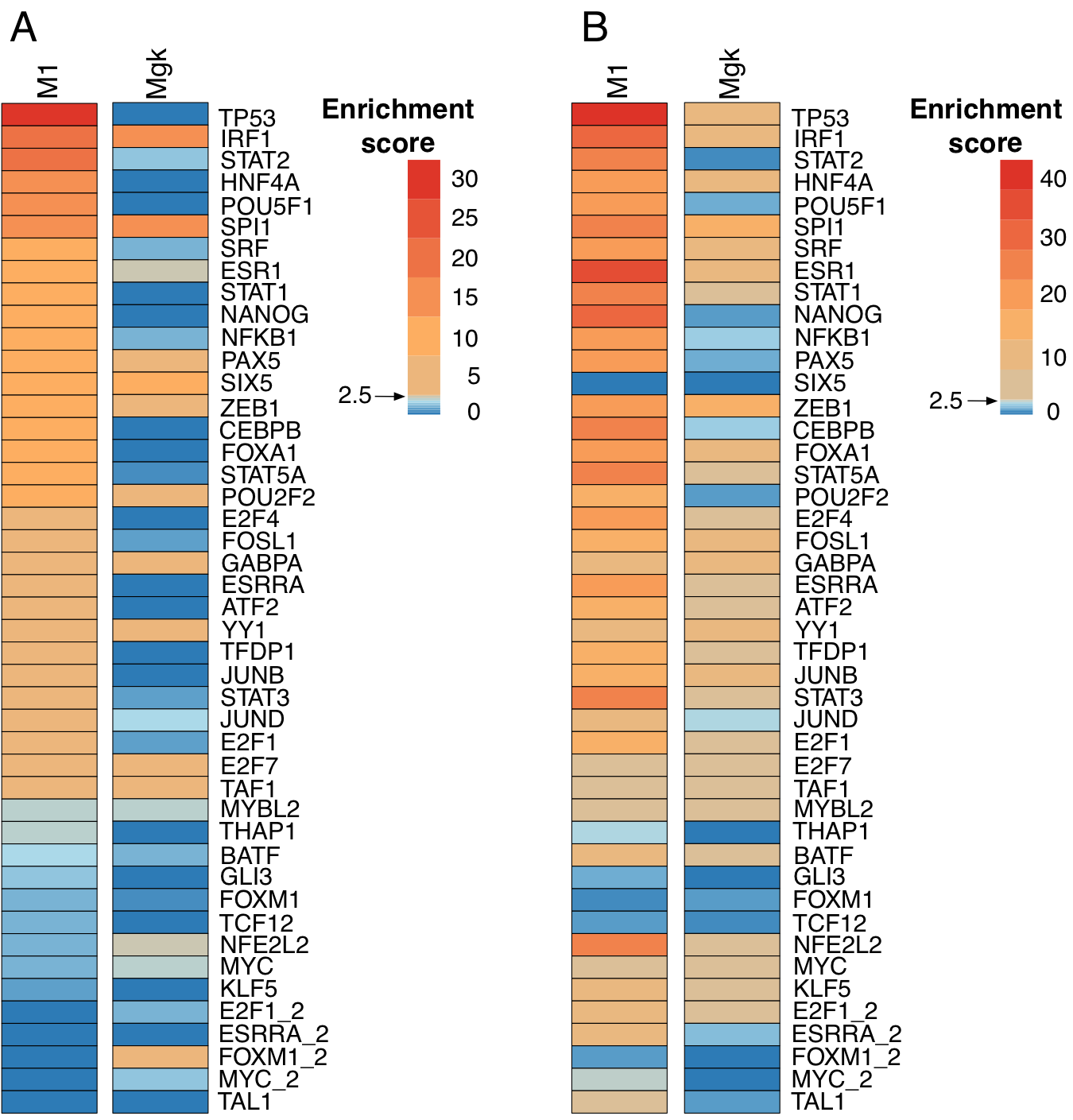

Supplement: S19 Fig — After genome-wide CRM scoring, removing the training CRMs, evaluating the enrichment of ChIP-seq peaks of the corresponding TF, and the enrichment of motifs of the corresponding TF, within the top 1000 newly predicted CRMs. Enrichment is represented as a Normalized Enrichment Score (NES) calculated by i-cisTarget. A) Significant enrichment of ChIP-seq peaks (orange is NES>2.5) for 31/45 M1 models, compared to 12/45 of the Mgk models. B) The motif of the respective TF is also enriched in the top 1000 newly predicted functional CRMs, for those in orange (NES>2.5). (TIFF) [file pcbi.1004590.s019.tiff]

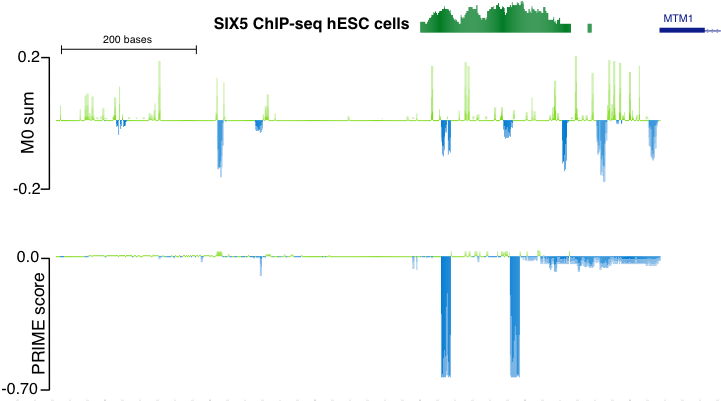

Supplement: S20 Fig — For the “M0 sum” model we summed the maximal motif scores (using the same PWMs as for M1 model) found in the 900bp regions upstream of MTM1 gene. Possible nucleotide substitutions demonstrate that M1 PRIME score are more specific and most scoring nucleotides are within the ChIPed region, which is not the case for M0 sum model. (TIFF) [file pcbi.1004590.s020.tiff]

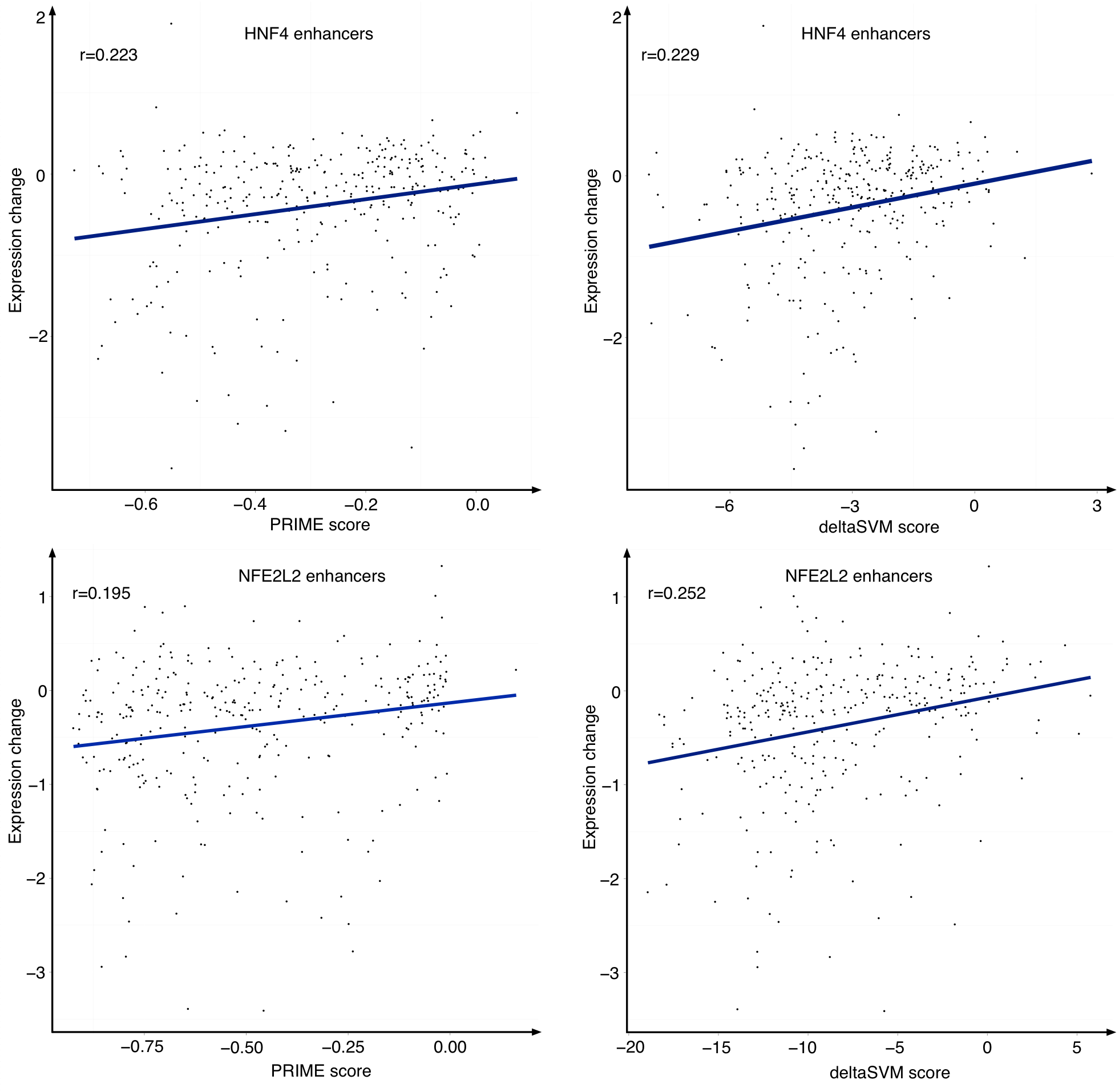

Supplement: S21 Fig — M1 and deltaSVM models (trained on the same sequences for NFE2L2 and HNF4A TFs) show association of the delta scores (predicted impact, x-axis) with reporter expression changes (y-axis). Both methods demonstrate comparable performance. (TIFF) [file pcbi.1004590.s021.tiff]

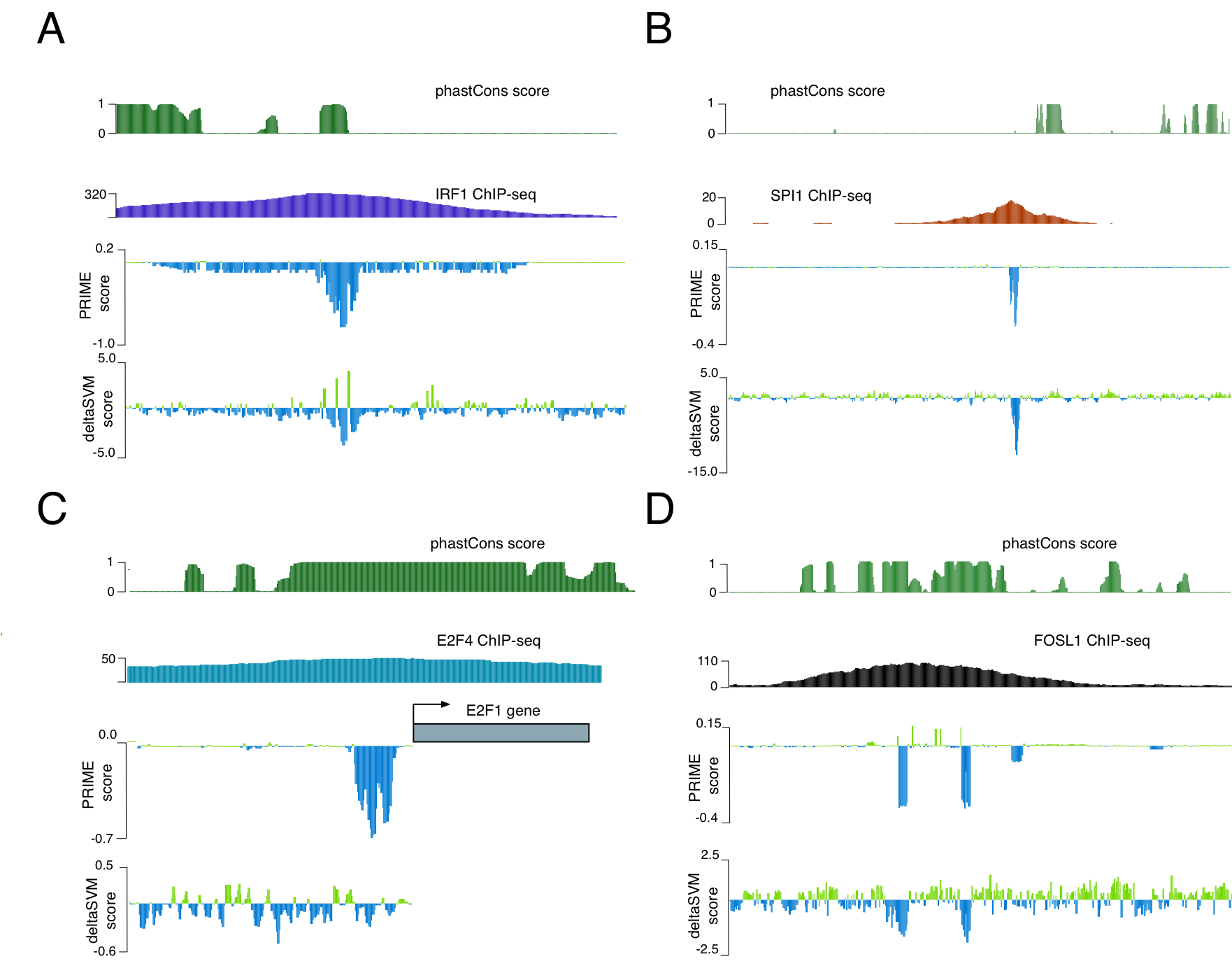

Supplement: S22 Fig — M1 and deltaSVM models for IRF1, SPI1, E2F4 and FOSL1 models where applied to predict the impact of simulated nucleotide substitutions. Both methods demonstrate good agreement with each other identifying the highest scoring nucleotides within the ChIP’ped regions for IRF1 and SPI1 but E2F4 and FOSL1 models are more specific for the Random Forest M1 model than for deltaSVM. (TIFF) [file pcbi.1004590.s022.tiff]

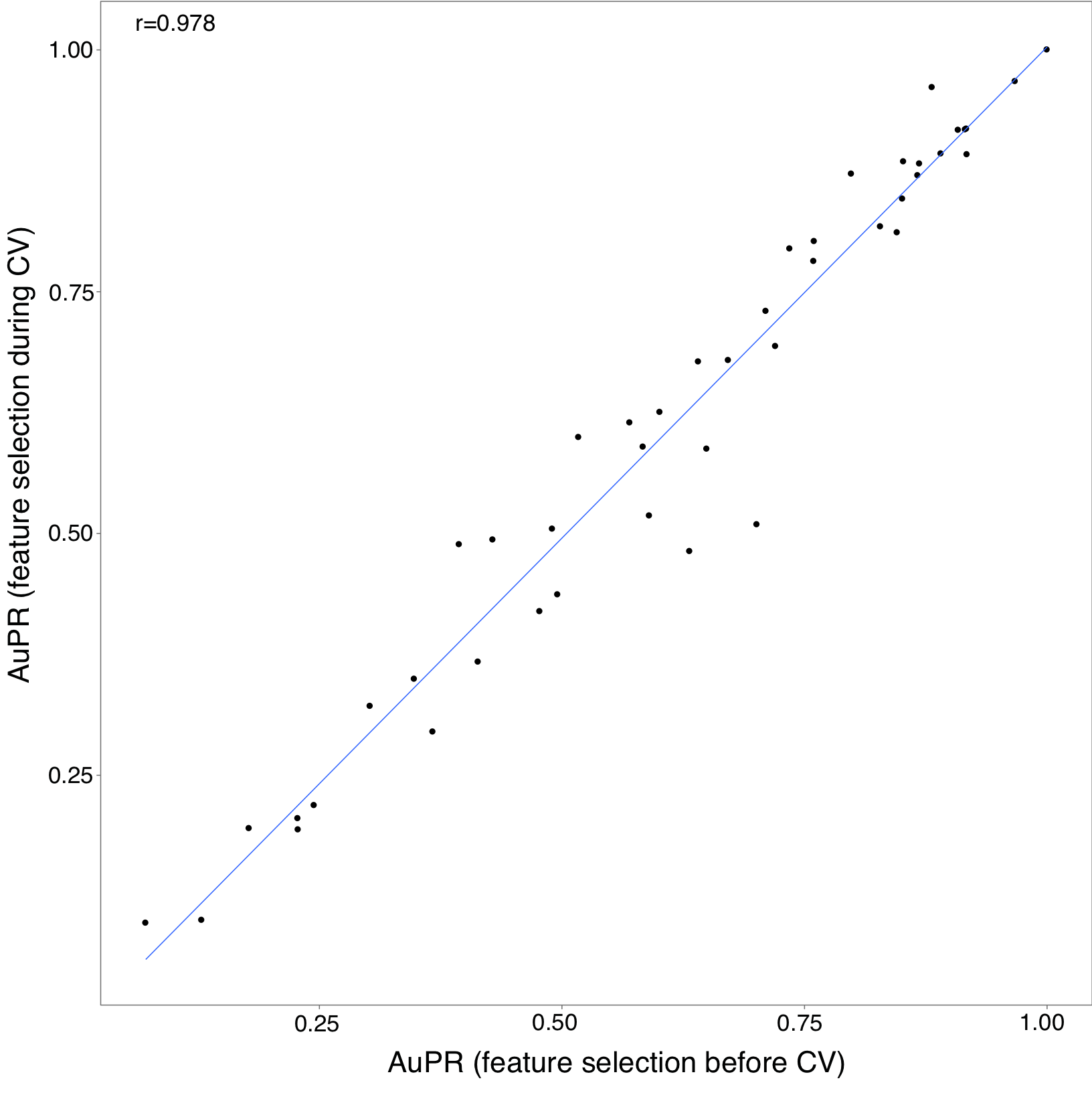

Supplement: S23 Fig — The performance of the M1 models using features selected on the entire dataset is comparable (r = 0.978) with models utilizing features identified only using training subset of the data and applied to the test data not participated in the selection. (TIFF) [file pcbi.1004590.s023.tiff]
